# G-site residue S67 is involved in the fungicide-degrading activity of a tau class glutathione *S*-transferase from *Carica papaya*

**DOI:** 10.1016/j.jbc.2024.107123

**Published:** 2024-02-28

**Authors:** Su-Yan Wang, Yan-Xia Wang, Sheng-Shuo Yue, Xin-Chi Shi, Feng-Yi Lu, Si-Qi Wu, Daniela D. Herrera-Balandrano, Pedro Laborda

**Affiliations:** School of Life Sciences, Nantong University, Nantong, China

**Keywords:** biodegradation, disulfide, enzyme catalysis, enzyme mechanism, food safety, fungicide degradation, glutathione peroxidase, plant detoxification system, plant molecular biology, site-directed mutagenesis

## Abstract

Thiram is a toxic fungicide extensively used for the management of pathogens in fruits. Although it is known that thiram degrades in plant tissues, the key enzymes involved in this process remain unexplored. In this study, we report that a tau class glutathione *S*-transferase (GST) from *Carica papaya* can degrade thiram. This enzyme was easily obtained by heterologous expression in *Escherichia coli*, showed low promiscuity toward other thiuram disulfides, and catalyzed thiram degradation under physiological reaction conditions. Site-directed mutagenesis indicated that G-site residue S67 shows a key influence for the enzymatic activity toward thiram, while mutation of residue S13, which reduced the GSH oxidase activity, did not significantly affect the thiram-degrading activity. The formation of dimethyl dithiocarbamate, which was subsequently converted into carbon disulfide, and dimethyl dithiocarbamoylsulfenic acid as the thiram degradation products suggested that thiram undergoes an alkaline hydrolysis that involves the rupture of the disulfide bond. Application of the GST selective inhibitor 4-chloro-7-nitro-2,1,3-benzoxadiazole reduced papaya peel thiram-degrading activity by 95%, indicating that this is the main degradation route of thiram in papaya. GST from *Carica papaya* also catalyzed the degradation of the fungicides chlorothalonil and thiabendazole, with residue S67 showing again a key influence for the enzymatic activity. These results fill an important knowledge gap in understanding the catalytic promiscuity of plant GSTs and reveal new insights into the fate and degradation products of thiram in fruits.

Fruits include dietary fiber, vitamins, and minerals, as well as a variety of health benefits (1, 2). Papaya (*Carica papaya*) is a tropical and subtropical fruit that is widely cultivated (3). This fruit is high in vitamins A, B9, and C, along with carotenoids and phenolic compounds, and has anticancer properties (4, 5). However, papaya fruit is susceptible to a range of fungal necrotrophic pathogens that cause black spots on the fruit surface, hindering its commercialization (6, 7).

The control of fungal pathogens strongly relies on the application of fungicides, which are considered indispensable to secure fruit supply (8, 9). Thiram, tetramethylthiuram disulfide (1), is a broad-spectrum fungicide commercialized by Bayer (Fig. S1). Despite 1 being thoroughly used for the control of diseases in fruits worldwide (10), it can contaminate the environment and has shown numerous toxic effects on animals, including cell mitochondrial damage, apoptosis, hyperglycemia, neurodegeneration, and infertility (11, 12). This fungicide is also used in papayas to avoid pathogenic infections (13) and has been reported to show low diffusion ability in fruits, remaining in the fruit peel (14). It has been indicated that 1 degrades in aqueous solution to dimethyl dithiocarbamate (2) and, subsequently, to triethylamine (3) and carbon disulfide (4) (Fig. S2); however, the degradation is slow, with half-lives of 7.8 and 5 days at 20 °C and 30 °C, respectively (15). Although a few approaches for the decontamination of 1 from aqueous solutions have been reported (16), methods to decontaminate 1 in fruits are lacking.

Glutathione *S*-transferases (GSTs) are enzymes present in animals, plants, and some microorganisms, playing a key role in the GSH network of oxidative stress defense (17). GSTs are the main component in the detoxification system against damage and cancer in arthropods and mammals, respectively (18). The key role of some GSTs in cancer progression has stimulated the development of selective GST inhibitors, such as 4-chloro-7-nitro-2,1,3-benzoxadiazole (NBD-Cl), for pharmaceutical use (19). Plant genomes contain between 20 and 100 genes encoding GSTs (20, 21). Plant GSTs are grouped into five classes, including phi, zeta, tau, theta, and lambda, based on sequence identity and gene organization, and the tau and phi class GSTs appear to be unique to plants (22). Twenty-six GSTs have been predicted in the papaya genome, with most of them belonging to the tau-class (Table S1) (23). Plant GSTs are known to play major roles in oxidative stress metabolism toward abiotic stresses, such as drought and salt stresses (24, 25). GSTs are involved in the detoxification of arsenic (26) and the biosynthesis and subcellular sequestration of specialized metabolites (27, 28) and have been reported to participate in plant-induced systemic resistance response to pathogen infection (29).

GSTs are promiscuous enzymes capable of catalyzing different reactions; however, they often function as GSH peroxidases, oxidizing GSH in the presence of peroxides to connect two glutathione tripeptides *via* a disulfide bond or conjugating GSH to a xenobiotic (30). Some reports have indicated that the G-site residue S13 is required for GSH oxidation, suggesting that this residue plays a key role in the construction of the disulfide bond (31, 32, 33). Although this residue is present in the phi, zeta, tau, and theta class GSTs, lambda GSTs contain an active cysteine residue in the same site (34, 35). A few reports have indicated that GSTs can degrade pesticides and herbicides. For example, the GST from *Klebsiella jilinsis* was reported to degrade chlorimuron-ethyl by breaking the amide bonds contained in the structure (36), while the GST from *Gordonia rubripertincta* CWB2 catalyzed the addition of styrene epoxide to GSH (37). In addition to the GSTs from bacterial sources, Deng *et al*. predicted that the tau class GSTs from jujube were involved in the degradation of various pesticides, including malathion and glyphosate (38), while the tau class GSTs from rice were reported to degrade chloro-S-triazine and acetanilide (39). Although GSTs have been shown to degrade organic compounds with very different functional groups, the reaction mechanisms and active site residues involved in the reactions remain unknown.

Given that the natural reaction of GSTs is to form disulfide bonds with GSH as substrate and 1 contains a disulfide bond, we considered that 1 could be a potential substrate for GSTs. This study aimed to evaluate the ability of a tau class GST from papaya (TCpGST; UniProt accession number: O49821), which is highly expressed during fruit ripening (40) to degrade 1. The findings of this study revealed new insights on GST promiscuity and the degradation products of 1 in fruits.

## Results and discussions

### TCpGST can be expressed in *E. coli*

**Figure 1:**
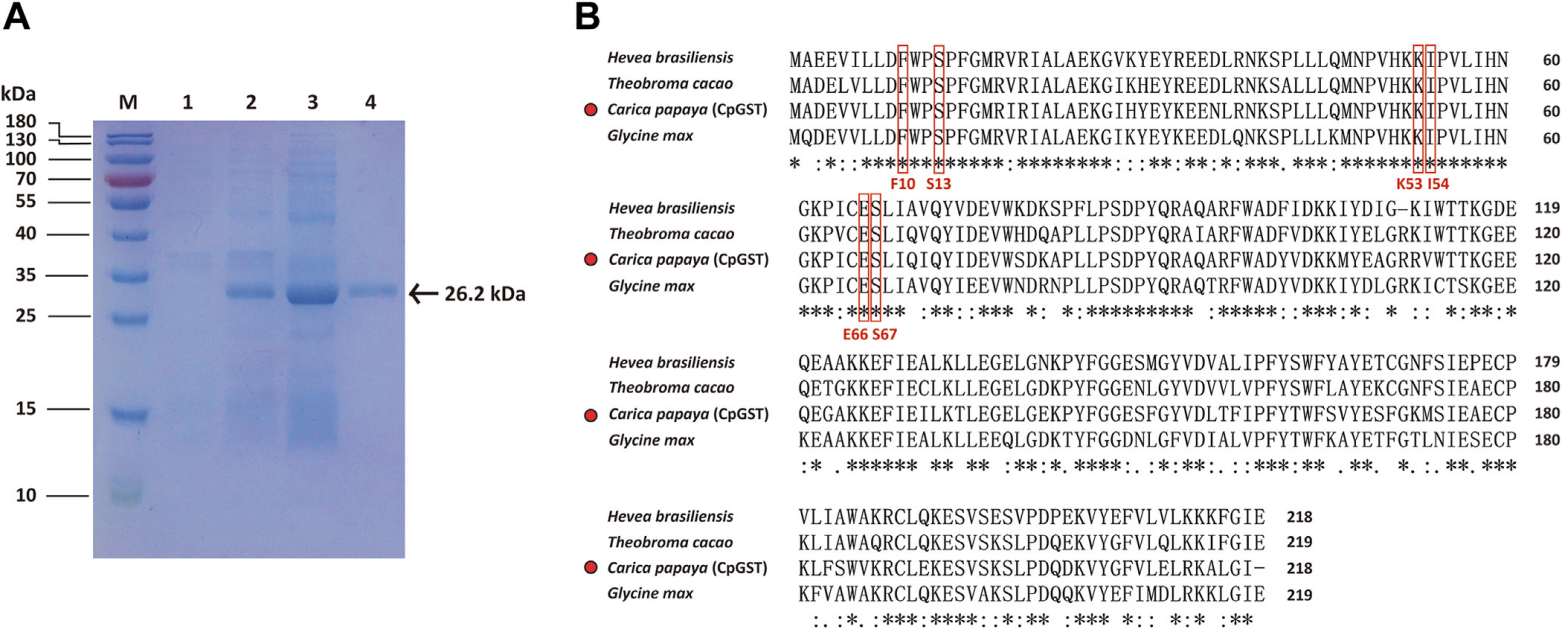
**SDS-PAGE analysis of TCpGST and amino acid sequence alignment.***A*, SDS-PAGE analysis of recombinant TCpGST containing an additional C-terminal hexa-histidine tag, after staining with Coomassie brilliant blue G-250. M, protein marker; 1, cell pellets before induction; 2, cell pellets after induction; 3, supernatant of cell lysate; and 4, Ni-NTA purified enzyme (0.05 μg). *B*, amino acid sequence alignment of TCpGST and some representative tau class GSTs. Fully conserved amino acids are labeled with *asterisks*, conserved substitutions with *colons*, and semiconserved substitutions with a *dot*. GST, glutathione *S*-transferase; Ni-NTA, Ni (II)-nitrilotriacetate agarose; TCpGST, tau class glutathione *S*-transferase from *Carica papaya*.

The nucleotide sequence registered in GenBank (GenBank accession number: XP_021895014) was used to design the primers to amplify the putative *TCpGST* gene. The full-length ORF of *TCpGST* could be successfully cloned and consisted of 657 base pairs (Fig. S3). The putative gene products could be expressed by *E. coli* in soluble form containing an additional *C*-terminal hexa-histidine tag (Fig. 1*A*). SDS-PAGE analysis showed a dominant protein band of the expected molecular weight (26.2 kDa) after Ni (II)-nitrilotriacetate (Ni-NTA) agarose affinity chromatography.

BLAST analysis showed that TCpGST had 79.36%, 79.36%, 76.16%, and 77.06% homology to the GSTs from *Theobroma cacao* (GenBank accession number: EOY13724), *Prunus dulcis* (GenBank accession number: XP_034213852), *Glycine max* (GenBank accession number: 2VO4_A), and *Vitis vinifera* (GenBank accession number: XP_002262767), respectively. TCpGST showed high identity with respect to several reported tau class GSTs, such as *T. cacao* GST tau19 (GenBank accession number: EOY13724), *Hevea brasiliensis* GST tau1 (GenBank accession number: AEF79856), and *G. max* tau class GST (GenBank accession number: 5AGY_A), showing numerous conserved domains compared to these representative tau class GSTs (Fig. 1*B*, and Fig. S4).

### TCpGST degrades thiram under physiological reaction conditions

**Figure 2:**
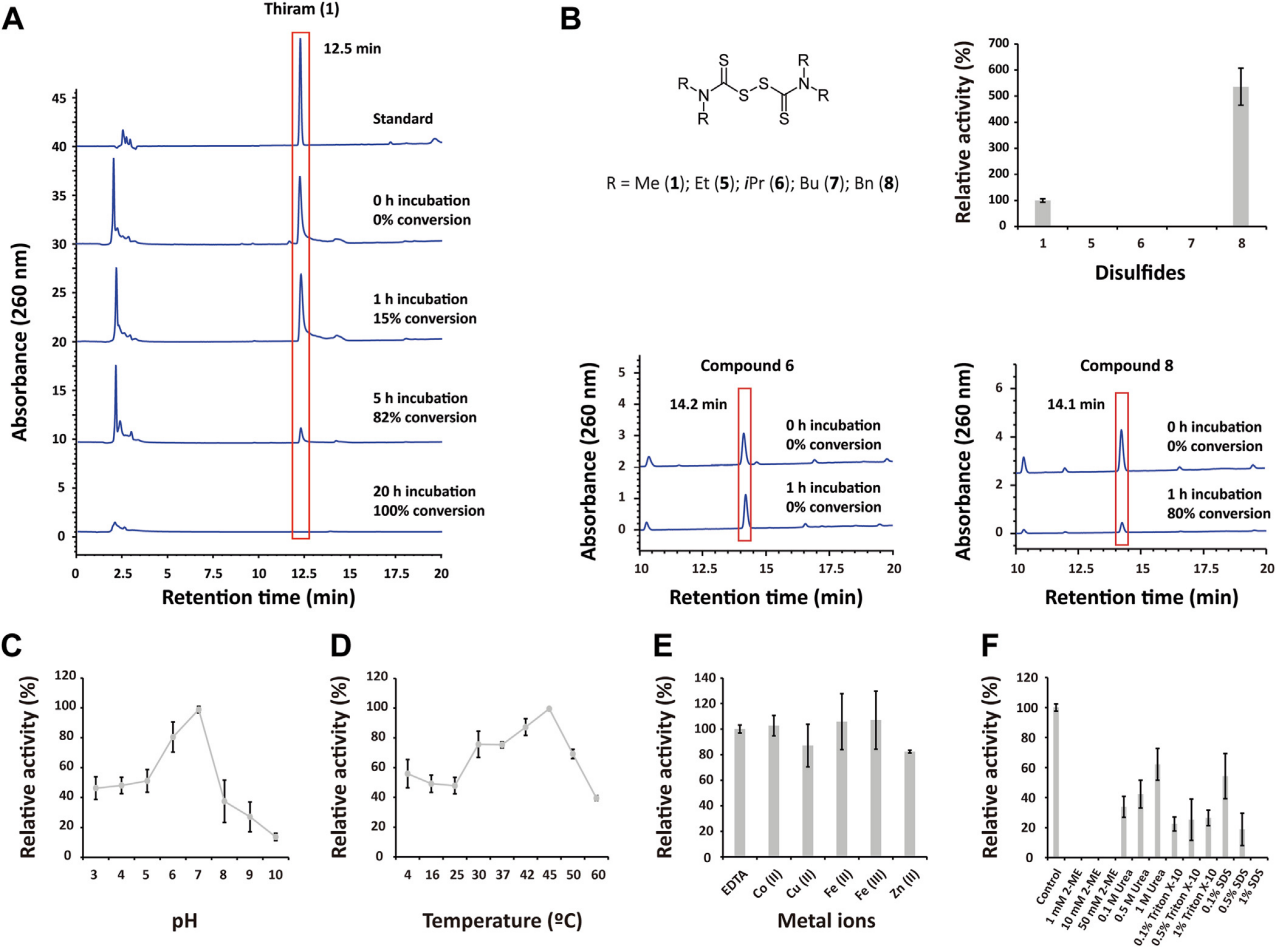
**Activity and biochemical properties of TCpGST toward thiram (1).***A*, HPLC-based analysis of TCpGST-catalyzed degradation of 1. The reaction system contained 0.2 mM 1 and 0.01 U TCpGST in 50 mM Tris buffer (total volume = 50 μl) and was shaken at 200 rpm and 28 °C. The reaction was stopped by adding 50 μl methanol. The degradation of 1 was monitored using an HPLC system equipped with a C18 column at 260 nm. The mobile phase (1 ml/min) consisted of a gradient from 30% to 77% acetonitrile from 0 to 20 min. *B*, promiscuity of TCpGST toward disulfides tetraethylthiuram (5), tetraisopropylthiuram (6), tetrabutylthiuram (7), and tetrabenzylthiuram disulfides (8). The reaction systems contained 0.2 mM disulfide and 0.01 U TCpGST in 50 mM Tris buffer (total volume = 50 μl) and were shaken at 200 rpm and 28 °C for 1 h. The degradation of the disulfides was monitored using an HPLC system equipped with a C18 column at 260 nm. *C*, pH-dependence of recombinant TCpGST. pH values ranged from 3 to 10. The reactions were carried out at 30 °C. *D*, temperature dependence of recombinant TCpGST. The screened temperatures ranged from 4 to 60 °C. The reactions were carried out at pH 6. *E*, effect of metal ions on TCpGST activity. The reactions were performed in the presence of either 1 mM EDTA or 1 mM Co (II), Cu (II), Fe (II), Fe (III), and Zn (II). All metals were added to the reaction in their chloride form. The reactions were carried out at 30 °C and pH 6. *F*, effect of 2-mercaptoethanol (2-ME; 1, 10, and 50 mM), SDS; 0.1%, 0.5%, and 1% *v/v*, Triton X-100 (0.1%, 0.5%, and 1% *v/v*), and urea (0.1, 0.5, and 1 M) on TCpGST activity. TCpGST, tau class glutathione *S*-transferase from *Carica papaya*.

To confirm the degrading activity of recombinant TCpGST toward 1, the concentration of 1 was monitored at different time points in the presence and absence of the enzyme. The peak corresponding to 1 decreased in the HPLC chromatogram, confirming that TCpGST can degrade 1 (Fig. 2*A*). The degradation rate was 15% after 1 h, while no peak was detected after 20 h reaction time (100% conversion). In the absence of TCpGST, the concentration of 1 did not change after incubation for 1 h, while the concentration of 1 slightly decreased by 6% after incubation for 20 h. The *K*_m_ value of TCpGST for 1 was 0.1484 ± 0.007 mM, the *V*_max_ was 0.1004 ± 0.003 mM/min, and the *k*_cat_ value was 23.602 ± 0.003 min^−1^ (Table S2). Although the degradation of 1 in plants and fruits has been previously reported (41), this is the first report that confirms the degrading activity of plant tau class GSTs toward 1. Given that tau class GSTs are present in most plants; this result provides new insights into the plant agents that can degrade 1. It must be noted that GSTs oxidize GSH in the presence of peroxides (17), but the presence of peroxides was not required for the degradation of 1, suggesting that both reactions involve different mechanisms.

To understand the promiscuity of TCpGST toward other disulfides, fungicides 5, 6, 7, and 8 were used as substrates (Fig. 2*B*). All used fungicides consisted of thiuram disulfide structures bearing different alkyl groups connected to the amine groups. Interestingly, TCpGST showed 25% higher catalytic efficiency (*k*_cat_/*K*_m_) toward 8 than 1 (Table S2). The *V*_*max*_ of TCpGST toward 8 was 419.5-fold higher than toward 1, indicating that TCpGST can catalyze the degradation of 8 much faster than the degradation of 1. However, no catalytic activity was detected when using disulfides 5, 6, and 7 as substrates.

Temperature-, pH-, and metal-dependency of TCpGST and the effects of various additives on TCpGST activity were examined using 1 as substrate. The highest activities were measured at pH 7 and 45 °C (Fig. 2, *C* and *D*). The activity was maintained from 4 °C to 60 °C. The effect of metals on TCpGST activity was investigated by carrying out the reaction in the presence of various metal ions or EDTA. The results suggested that the enzymatic activity was not dependent on metal ions (Fig. 2*E*). TCpGST activity was slightly inhibited by Zn (II) (1 mM concentration), while Co (II), Cu (II), Fe (II), and Fe (III) did not cause any inhibitory effect.

The addition of 2-mercaptoethanol and detergents Triton X-100 and SDS involved large inhibitory effects on TCpGST activity (Fig. 2*F*). The inhibitory effects were especially great in the presence of 2-mercaptoethanol and SDS, which inhibited completely TCpGST activity at 1 mM and 1% *v/v* concentrations, respectively. In contrast, urea slightly enhanced the enzymatic activity.

### Residue S67 is necessary for the thiram-degrading activity of TCpGST

The pockets from tau class GSTs have been thoroughly studied. The active site of a tau class GST from *G. max* (also called G-site) was identified using GSH as substrate, and the potential catalytic residues, including F10, S13, K53, I54, E66, and S67, were predicted (42, 43). As can be seen in Fig. 1*B*, those residues are conserved in the tau class GSTs throughout the species. Cummins *et al*. indicated that residues E66 and S67 showed electrostatic interactions with GSH in the active site of the tau class GSTs from *Arabidopsis thaliana*, *Triticum aestivum*, and *Zea mays* (44). As indicated in the introduction section, it has been reported that the residue S13 is essential for the formation of the disulfide bond to connect two GSH molecules in the plant phi and theta class GSTs (31, 32, 33) and mutation of this residue to alanine in a *Lucilia cuprina* theta class GST inactivates the enzyme (45). The mutation of the S13 residue in *Oryza sativa* OsGST3 and OsGST5 reduced almost completely the activity of the enzymes toward GSH (33, 46).

**Figure 3:**
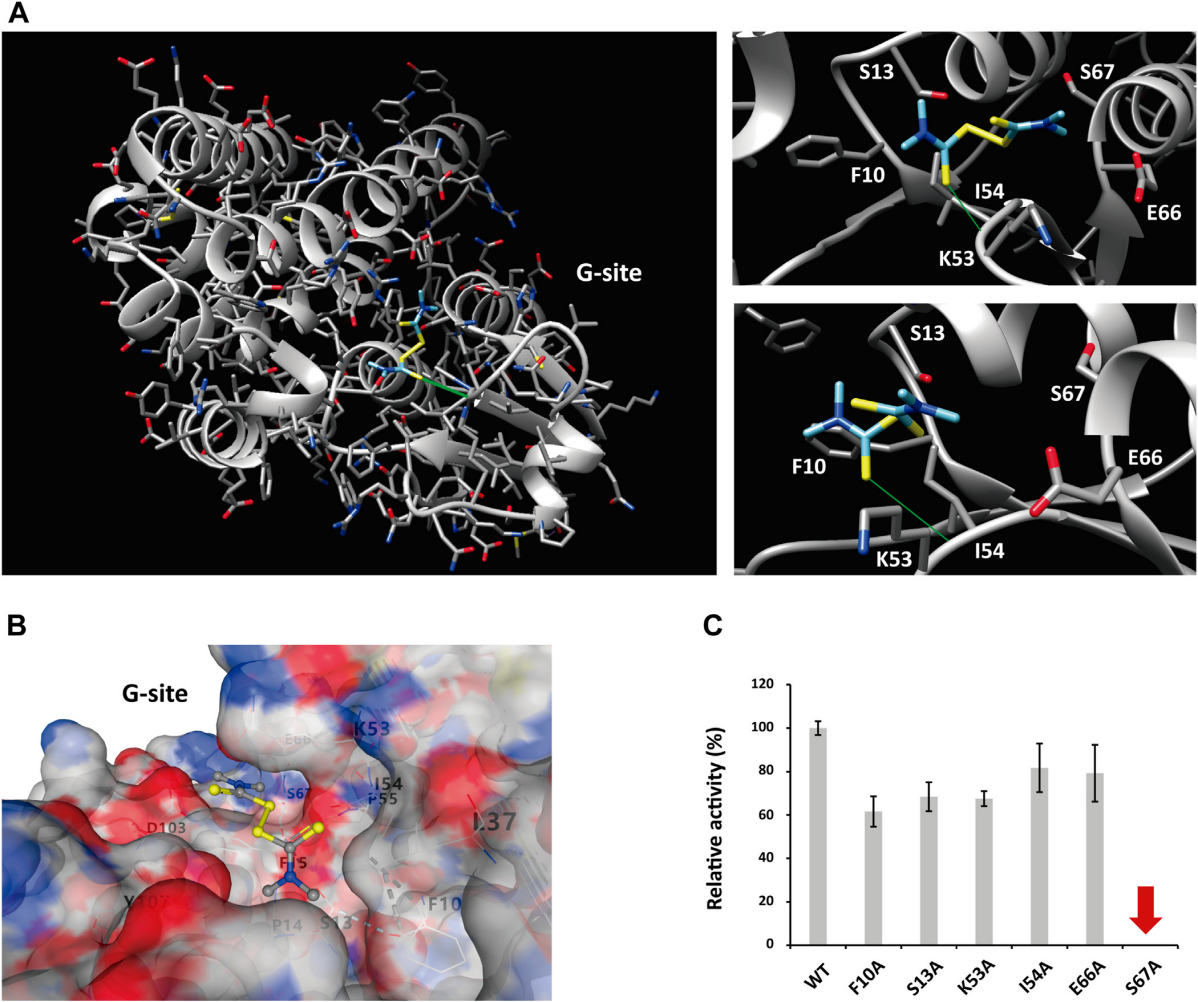
**TCpGST structural analysis and residues involved in the enzymatic reaction.***A*, interactions of thiram (1) with the residues in the active site of TCpGST. The three-dimensional structure of TCpGST was predicted using Phyre2 according to the reported X-ray three-dimensional structure of the tau class GST from *Glycine max* (https://www.ebi.ac.uk/pdbe/entry/pdb/2vo4) (28). The docking study was performed using SwissDock (56). *B*, conformation of 1 in the G-site of TCpGST. The location of 1 in the G-site of TCpGST was calculated using the CB-Dock software (55). *C*, activity study of WT and TCpGST active site mutants toward 1. The mutants were constructed by replacing residues F10, S13, K53, I54, E66, and S67 with alanine. Mutant S67A did not show degrading activity toward 1. TCpGST, tau class glutathione *S*-transferase from *Carica papaya*.

In agreement with previous studies, the minimum energy conformation for 1 was detected in the G-site of TCpGST (Fig. 3, *A* and *B*). It must be noted that diphenyl ether herbicides have been reported to have the lowest energy conformations in the G-site of the tau class GST from *G. max* (31), suggesting that this site may be the main site responsible for the degradation of organic substances. Docking studies revealed high proximity between the residues F10, S13, K53, I54, E66, and S67 and 1 in the active site of TCpGST (energy = −20.4182). The sulfur atom of one of the thioketones of 1 showed a strong polar interaction with the amide of I54. The distances between F10, S13, K53, I54, E66, and S67 radical chains and the disulfide bond of 1 were 8.279, 6.363, 5.766, 4.209, 8.262, and 9.761 Å, respectively. Docking of 8 in TCpGST indicated that this compound also shows the lowest energy conformation in the TCpGST G-site and shows high proximity to the same residues and residue F15 (Fig. S5).

To examine the participation of residues F10, S13, K53, I54, E66, and S67 in the degradation of 1, mutant enzymes were constructed by replacing these residues with alanine (Figs. S6 and S7, and Table S3). Mutant S67A showed no activity (Fig. 3*C*), indicating that S67 may be the catalytic residue responsible for the degradation of 1. Previous reports using phi and theta class GSTs and GSH as substrate indicated that the enzymatic activity was only reduced when mutating the residue S13, but not the residue S67 (32, 33). This result can be explained by considering that different substrates may show different interactions with the G-site residues. Although the residue S13 is replaced by cysteine in the lambda class GSTs, the residue S67 remains conversed across the different GST classes (Figs. S8 and S9). The catalytic efficiency (*k*_cat_/*K*_m_) of F10A, S13A, K53A, I54A, and E66A was lower than TCpGST (Table S4), suggesting that these residues probably show interactions with 1 and stabilize the substrate in the G-site but are not the catalytic residues toward 1.

Interestingly, the catalytic efficiency (*k*_cat_/*K*_m_) of S13A toward GSH was 11.56 times lower than that detected when using the WT protein (Table S5). Instead, the catalytic efficiency was only reduced by 27%, 25%, 48%, 51%, and 30% when using mutants F10A, K53A, I54A, E66A, and S67A, respectively. The different activities of the mutant enzymes toward 1 and GSH provide conclusive evidence for the existence of at least two catalytic residues, S13 and S67, in the G-site.

### TCpGST degrades thiram into carbon disulfide and dimethyl dithiocarbamoylsulfenic acid

**Figure 4:**
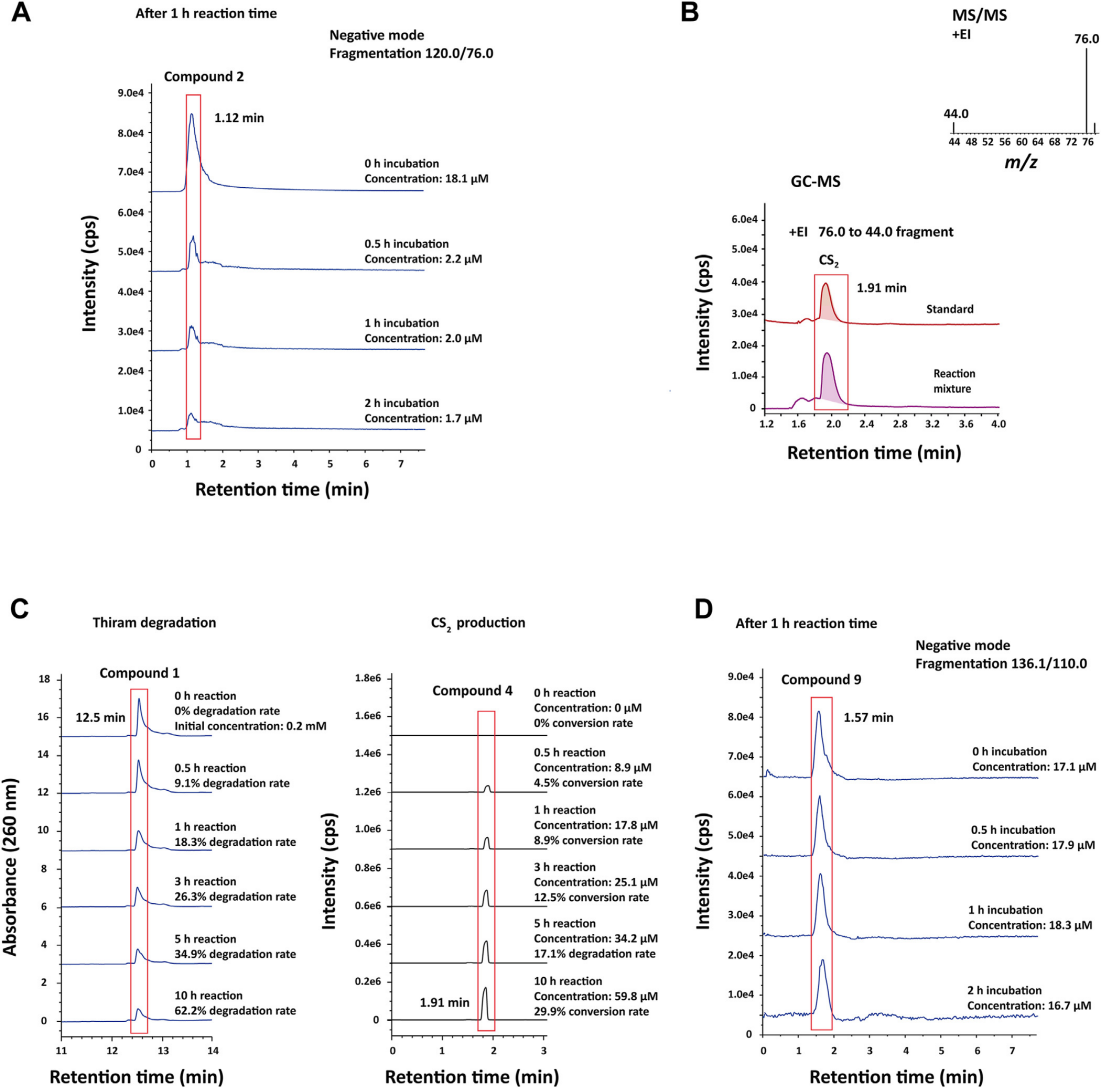
**Analysis of thiram (1) degradation products.***A*, LC-MS analysis of dimethyl dithiocarbamate (2) stability. The reaction system contained 0.2 mM 1 and 0.01 U TCpGST in 50 mM Tris buffer (total volume = 50 μl) and was shaken at 200 rpm and 28 °C for 1 h. The reaction was stopped by adding 50 μl methanol. The concentration of 2 was monitored at 0, 0.5, 1, and 2 h after stopping the reaction. 2 was detected using an LC-MS instrument equipped with an Agilent SB-C18 column (21 × 75 mm) at 25 °C following the conditions reported by Gupta *et al*. (47). The concentration of 2 decreased over time confirming its degradation. *B*, GC-MS analysis of carbon disulfide (4) as a product of TCpGST-catalyzed degradation of 1. The reaction was scaled up to 500 μl reaction volume using a 20 ml sealed glass tube. After stopping the reaction with methanol (500 μl), the solution was kept at 28 °C for 2 h, and 1 ml of the headspace gases was injected in a GC-MS instrument. The ratio in positive mode for the 76/78 *m/z* peaks was 10:1, which agrees with previously reported *m/z* peaks of 4 (48, 49). The main *m/z* fragment of 76.0 was detected at 44.0. *C*, comparison of 1 degradation and simultaneous production of 4. The concentration of 4 was measured after stopping the reaction and incubating the reaction mixture for 2 h. The conditions indicated in the previous sections were used to monitor the degradation of 1 and the formation of 4. The concentration of 4 was approximately half the degradation of 1. *D*, LC-MS analysis of dimethyl dithiocarbamoylsulfenic acid (9) stability. The enzymatic degradation of 1 was conducted as described in section *A*. The reaction was stopped after 1 h, and the concentration of 9 was monitored at 0, 0.5, 1, and 2 h after stopping the reaction. 9 was detected using an LC-MS instrument equipped with an Agilent SB-C18 column (21 × 75 mm) at 25 °C following the conditions reported by Gupta *et al*. (47). The concentration of 9 remained stable over time. TCpGST, tau class glutathione *S*-transferase from *Carica papaya*.

To propose TCpGST reaction mechanism toward 1, the reaction products obtained during the degradation of 1 were analyzed. Interestingly, 2 was detected in the reaction solution. This compound was detected in negative mode at 120.0 Da (calculated for C_3_H_6_NS_2_, [M ‒ H]^‒^ = 119.9942), which provided a main *m/z* peak at 76.0 Da after tandem mass spectrometry (MS/MS) fragmentation (Fig. S10). These results are consistent with the previously reported MS/MS analysis of 2 (47). Additionally, the ^1^H NMR spectrum showed two singlets at 3.40 and 3.37 ppm, corresponding to the rotamers of the methyl groups (Fig. S11); whereas the ^13^C NMR spectrum showed signals at 214.39 (C=S) and 43.89 (CH_3_) ppm (Fig. S12). The fragmentation from 120.0 to 76.0 was used in the LC-MS analysis for quantification. After stopping the reaction, the concentration of 2 rapidly decreased over time, indicating that 2 was unstable in the reaction medium (Fig. 4*A*). The concentration of 2 was reduced to 0.094-fold after stopping the reaction and incubating the resulting solution at 28 °C for 2 h. As indicated in the introduction section, 2 can be degraded in aqueous solution to 3 and 4, which can be explained by considering a possible intramolecular rearrangement that result in the cleavage of the N-C bond (15).

To confirm the degradation of 2 into 4, the concentration of 4 was analyzed by GC-MS (Fig. 4*B*). The ratio in positive mode for the 76/78 *m/z* peaks was 10:1, which agrees with the previously reported *m/z* peaks of 4 (48, 49). The main *m/z* fragment of 76.0 was detected at 44.0, which may correspond to the rupture of one of the C=S bonds. As expected, the concentration of 4 increased over time, while the concentration of 1 decreased. When stopping the reaction and analyzing the degradation products after 2 h at 28 °C, the degradation rate of 1 over time was found to be approximately double the conversion to 4 (Fig. 4*C*). This suggested that 50% of 1 is degraded to 2 and, subsequently, to 4, but there must be another degradation product that also accounts for 50% of the conversion. Abakerli *et al*. reported the presence of 4 in papaya peel after treating papaya fruit with 1 (50), which is consistent with the obtained results.

Considering the key role of S67 in the TCpGST active site and that this residue contains a hydroxy group in the radical chain, a possible explanation for TCpGST-catalyzed degradation of 1 was that the S67 hydroxy group was attacking the disulfide bond, inducing the breakage of the disulfide bond. A similar reaction mechanism was reported for the alkaline hydrolysis of 1, which resulted in the formation of equimolar amounts of 2 and 9 (51). Interestingly, 9 was detected in the reaction mixture and its concentration (16.7‒18.3 μM) was similar to that of 4 (17.8 μM) after 1 h reaction time. As in the case of 4, the concentration of 9 corresponded to 50% of the degradation of 1, suggesting that 9 is the other degradation product of 1. Compound 9 provided a main *m/z* peak at 138.0 Da in positive mode (calculated for C_3_H_8_NOS_2_, [M + H]^+^ = 138.0047), which provided fragments at 89.8 and 60.1 Da in the MS/MS analysis, and a main *m/z* peak at 136.1 Da in negative mode (calculated for C_3_H_6_NOS_2_, [M ‒ H]^‒^ = 135.9891), which provided fragments at 110.0 and 64.0 Da in the MS/MS analysis (Fig. S13). The obtained mass spectrometry (MS) spectra are consistent with previously reported spectra of 9 (47). ^1^H NMR analysis showed the presence of two singlets at 3.48 and 3.36 ppm, corresponding to the OH and the methyl groups, respectively (Fig. S14). On the other hand, the ^13^C NMR spectrum revealed signals at 213.88 (C = S) and 44.01 (CH_3_) ppm (Fig. S15). The fragmentation from 136.1 to 90.0 was used for LC-MS–based quantification. In contrast with 2, the content of 9 remained stable for 2 h (Fig. 4*D*), suggesting that this last compound does not degrade in short periods. The detection of 2 and 9 as the products of TCpGST-catalyzed degradation of 1 is consistent with the report by Gupta *et al*. (47), who indicated that 2 and 9 were the main degradation products of 1 in radish and tomato plants, while water and soil degraded 1 to provide mainly 2 and oxidation products.

**Figure 5:**
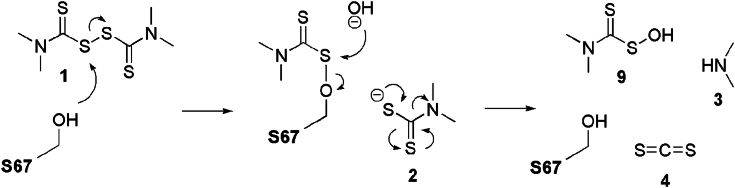
**Proposed enzymatic mechanism of TCpGST toward thiram (1).** S67 can attack the disulfide bond of 1, promoting the release of dimethyl dithiocarbamate (2), which subsequently degrades to dimethylamine (3), and carbon disulfide (4). Then, a hydroxy may participate to form dimethyl dithiocarbamoylsulfenic acid (9). TCpGST, tau class glutathione *S*-transferase from *Carica papaya*.

These results suggested that the hydroxy group of S67 may be able to attack one of the sulfur atoms of the disulfide bond of 1, causing the rupture of the disulfide bond (Fig. 5). This may release 2, which subsequently degrades into 3 and 4. A hydroxy group may in turn attack the sulfur atom attached to S67, releasing one molecule of 9. The proposed mechanism explains the presence of both 2 and 9 in the reaction medium. As far as we know, this is the first experimental evidence of GST-catalyzed degradation of 1. The proposed mechanism also explains why the highest enzymatic activity of TCpGST was observed at pH 7.0, wherein the concentration of hydroxy groups must be higher than at acidic pH values. It must be noted that the proposed mechanism can help to understand how GSTs break compounds containing disulfide bonds. However, GSTs have also been reported to degrade other organic compounds that do not contain disulfide bonds, such as diphenylether, chlorimuron-ethyl, and 1,2-epoxy-3-(*p*-nitrophenoxy)propane (36, 37, 38, 39). The mentioned compounds contain ether groups or are substituted ureas.

### Phi class CpGST also shows thiram-degrading activity

**Figure 6:**
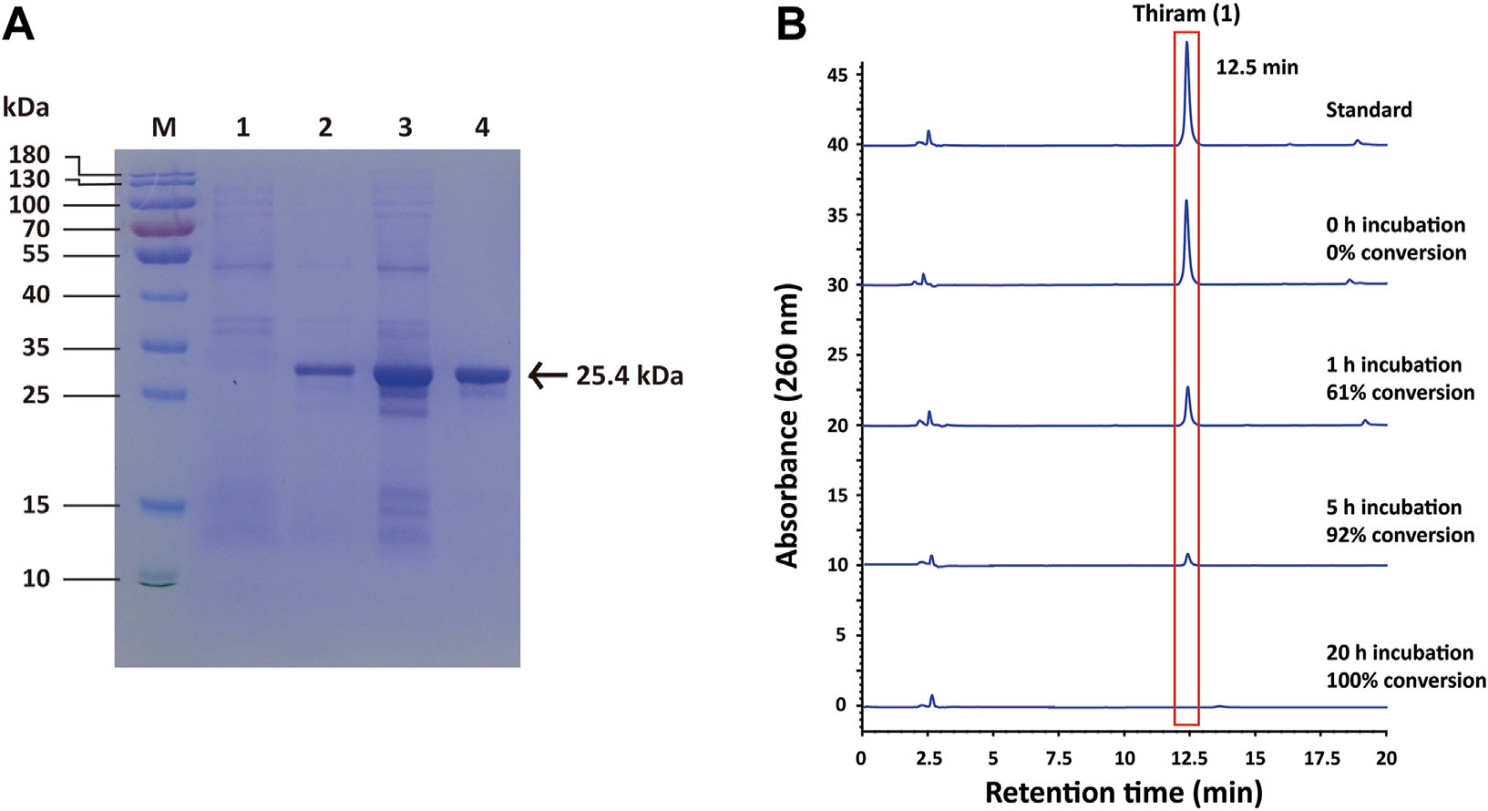
**SDS-PAGE analysis of PCpGST and activity toward thiram (1).***A*, SDS-PAGE analysis of recombinant PCpGST containing an additional C-terminal hexa-histidine tag, after staining with Coomassie brilliant blue G-250. M, protein marker; 1, cell pellets before induction; 2, cell pellets after induction; 3: supernatant of cell lysate; and 4, Ni-NTA purified enzyme (0.05 μg). *B*, HPLC-based analysis of PCpGST-catalyzed degradation of 1. The reaction system contained 0.2 mM 1 and 0.01 U PCpGST in 50 mM Tris buffer (total volume = 50 μl) and was shaken at 200 rpm and 28 °C. The reaction was stopped by adding 50 μl methanol. The degradation of 1 was monitored using an HPLC system equipped with a C18 column at 260 nm. The mobile phase (1 ml/min) consisted of a gradient from 30% to 77% acetonitrile from 0 to 20 min. Ni-NTA, Ni (II)-nitrilotriacetate agarose; PCpGST, phi class glutathione *S*-transferase from *Carica papaya*.

To understand the degradation ability of the papaya phi class GST (PCpGST; GenBank accession number: XP_021906879) and a papaya lambda class GST (LCpGST; GenBank accession number: XP_021891575), the corresponding enzymes were overexpressed in *E. coli* and their activity was screened. As shown in Table S1, eight lambda class GSTs and only one phi class GST have been predicted in the papaya genome. The nucleotide sequences registered in GenBank were used to design the primers to amplify the putative genes. The full-length ORFs of *PCpGST* and *LCpGST* could be successfully cloned and consisted of 639 and 618 base pairs, respectively (Fig. S16). The putative gene products could be expressed by *E. coli* containing an additional C-terminal hexa-histidine tag and purified *via* Ni-NTA agarose affinity chromatography (Fig. 6*A*, and Fig. S17).

BLAST analyses based on the amino acid sequences showed that PCpGST had 69.81% and 69.63% homology to the phi class GSTs from *Bruguiera gymnorhiza* (GenBank accession number: AEB77871) and *Hibiscus trionum* (GenBank accession number: GMI69406), respectively. LCpGST showed 76.11%, 60.20%, and 60.20% homology to the lambda class GSTs from *Populus trichocarpa* (GenBank accession number: ADB11342), *Larix kaempferi* (GenBank accession number: AHA46530), and *Pinus yunnanensi* (GenBank accession number: WAA68431), respectively.

When using PCpGST, the degradation rate of 1 was 61% after 1 h, while the peak corresponding to 1 was not detected after 20 h reaction time (100% conversion) (Fig. 6*B*), confirming that PCpGST can catalyze the degradation of 1. This result suggests that PCpGST may also contribute to the degradation of 1 in papaya. In contrast, LCpGST did not show any activity toward 1. The *K*_m_, *V*_max_, and *k*_cat_ values of PCpGST toward 1 were 0.0780 ± 0.0170 mM, 0.1095 ± 0.0119 mM/min, and 4741.8 ± 72.8 min^−1^, respectively (Table S6). Comparing the catalytic efficiency (*k*_cat_/*K*_m_) of TCpGST and PCpGST reveals that PCpGST exhibits 382.2-fold greater efficiency as catalyst than TCpGST.

Georgakis *et al*. reported that the phi class GSTs from *Hordeum vulgare* and *T. aestivum* displayed a broad range of specificity toward different thiols, conferring herbicide resistance in plants (52). Similarly, phi class GSTs were reported to play a key role in alachlor, atrazine, and chlorotoluron detoxification in *Alopecurus myosuroides* and *Lolium rigidum* (53). It has been indicated that behind tau class GSTs, phi class GSTs represent the second largest class in plants (54). Although lambda class GSTs have been reported to play important roles in enhancing plant tolerance to abiotic stresses, such as metals, cold, and salt (55), their ability to degrade organic substances has never been reported. Those previous reports appear to be consistent with the observed activity of TCpGST and PCpGST toward 1 and the lack of activity of LCpGST. Further experiments are necessary to examine the potential degrading activity of zeta and theta class GSTs.

### Papaya peel degrades thiram and the GSTs are the main degradation pathway

**Figure 7:**
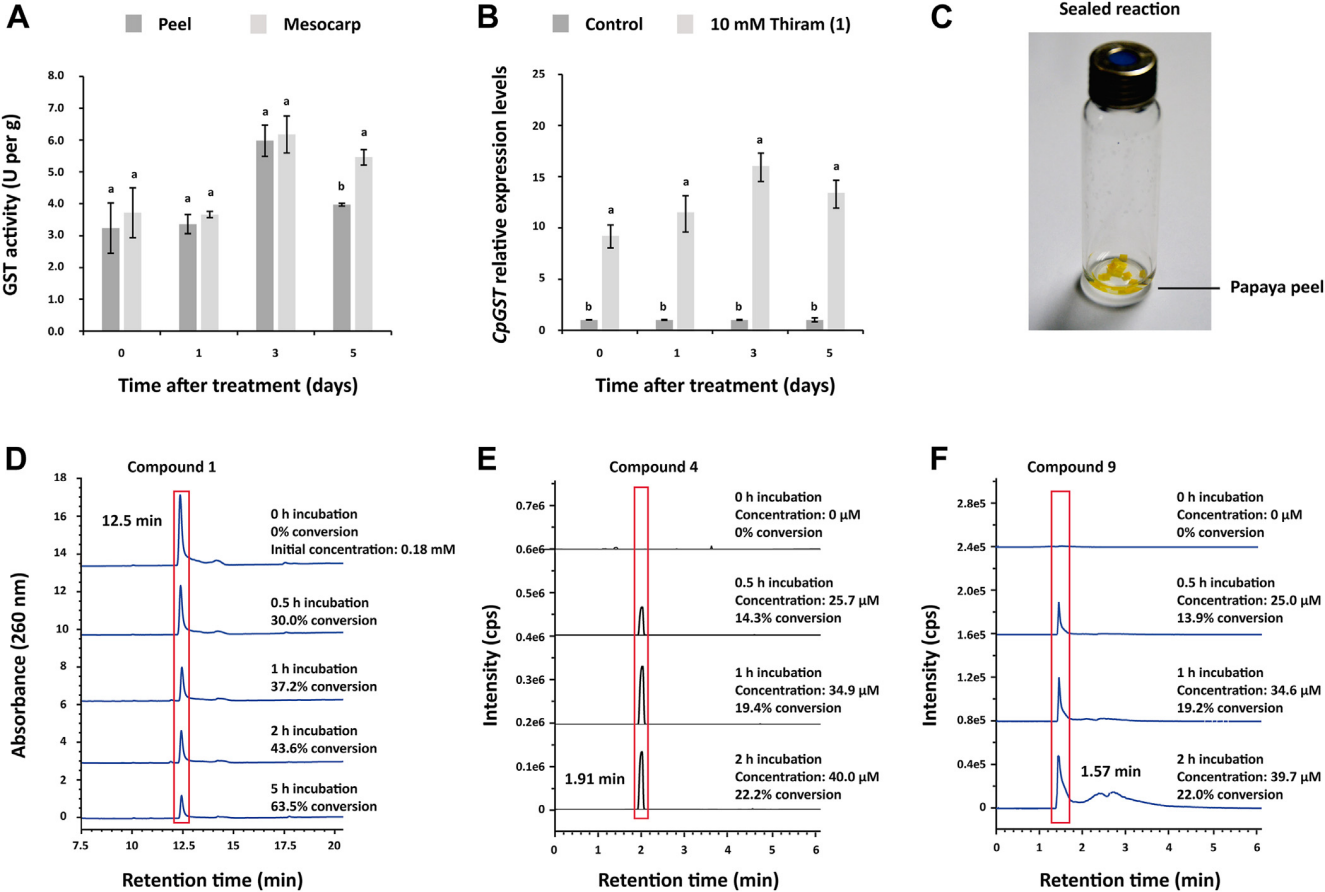
**Thiram (1)-degrading activity of papaya peel.***A*, GST activity of papaya peel and mesocarp. Papaya fruit was immersed in 400 ml aqueous solution containing 10 mM 1 for 20 min. GST activity was measured following the procedure described by Jo *et al*. (33). The enzymatic activity was evaluated using the changes in absorbance at 340 nm produced during the formation of 2,4-dinitrophenyl-glutathione from 1-chloro-2,4-dinitrobenzene and glutathione. Different letters above the bars indicate significant differences (*p* ≤ 0.05). *B*, *TCpGST* expression level in papaya peel after treatment with 10 mM 1 for 20 min. Real-time quantitative reverse transcription PCR was performed using a set of two PCR primers. *Actin* was used as the reference gene, and the relative gene expression was calculated by the 2^−ΔΔCT^ method. The control experiment was carried out in the absence of 1. Different letters above the bars indicate significant differences (*p* ≤ 0.05). *C*, images showing the reaction system using papaya peel as the catalyst in a sealed system. *D*, HPLC-based analysis of papaya peel–catalyzed degradation of 1. The reaction system consisted of 50 0.250 × 0.250 cm papaya peel pieces and 0.18 mM 1 (reaction volume = 500 μl). *E*, GC-MS-based analysis of carbon disulfide (4) as the degradation product of 1 in the presence of papaya peel. The reaction system consisted of 50 0.250 × 0.250 cm papaya peel pieces and 0.18 mM 1 (reaction volume = 500 μl). The content of 4 was estimated at 2 h after stopping the reaction with 500 μl methanol. *F*, LC-MS–based analysis of dimethyl dithiocarbamoylsulfenic acid (9) as the degradation product of 1 in the presence of papaya peel. The reaction system consisted of 50 0.250 × 0.250 cm papaya peel pieces and 0.18 mM 1 (reaction volume = 500 μl). GST, glutathione *S*-transferase; TCpGST, tau class glutathione *S*-transferase from *Carica papaya*.

GST activity was detected in both papaya peel and mesocarp after treating papaya fruit with 1. The enzymatic units per gram were similar in both papaya tissues. Although the GST activity did not change at 1 day posttreatment, the GST activity slightly increased after the first 3 days, decreasing after 5 days posttreatment (Fig. 7*A*). The GST activity in papaya peel was 3.24, 3.36, 5.98, and 3.92 U/g at 0, 1, 3, and 5 days posttreatment, respectively, confirming that papaya peel has GST activity. Interestingly, the application of 1 enhanced *TCpGST* expression levels in papaya peel, and the effect was detected just after the treatment with 1 (Fig. 7*B*). The mRNA levels of *TCpGST* were 9.1-, 11.8-, 15.0-, and 12.7-fold higher at 0, 1, 3, and 5 days posttreatment than those detected in the absence of 1. It must be noted that the highest GST activity and the highest expression of *TCpGST* were detected at 3 days posttreatment, indicating that both results follow the same trend. Previously, 1 was indicated to produce oxidative stress in tomatoes, promoting the accumulation of H_2_O_2_ and malondialdehyde (56). Thus, the increases in GST activity and *TCpGST* expression can be explained considering that 1 is causing an oxidative response, and papaya fruit increases GST activity to reduce the intracellular H_2_O_2_ concentration.

In agreement with the GST activity and the high expression levels of *TCpGST* found in papaya peel, this was found to degrade 1 (Fig. 7*C*). A 1.25 × 1.25 cm piece of papaya peel degraded 1 (0.18 mM) with 30.0%, 37.2%, 43.6%, and 63.5% conversion rates after 0.5, 1, 2, and 5 h, respectively (Fig. 7*D*). In agreement with the proposed reaction mechanism of TCpGST, 4 and 9 were detected in the reaction mixture using papaya peel as the catalyst, and their concentrations increased over time (Fig. 7, *E* and *F*). As expected, the concentrations of 4 (40.0 μM) and 9 (39.7 μM) were similar at 2 h reaction time (the stopped reaction was incubated at 28 °C for 2 h before the analysis of 4), and each one corresponded to approximately 50% of the amount of degraded 1. This result can be explained considering that papaya peel is expressing GSTs, including TCpGST, and these are transforming 1 into the mentioned compounds.

Although the toxicity of 9 has never been evaluated, 2 and 4 cause neurotoxic and hepatotoxic effects (57, 58). The boiling point of 4 is 46.3 °C, and its solubility in water is low (0.23 g in 100 ml at 22 °C), suggesting that this compound may remain in the fruit at common fruit growing and storage conditions, and after washing with water. This indicates that GST-catalyzed degradation of 1 is not removing completely its toxicity but producing other compounds that also show toxic effects. Although several research groups reported methods for the detection of 1 in fruits, and several countries have developed legislation to control its use and presence in food products, methods for detection, and appropriate regulations for controlling 2, 4, and 9 are lacking.

**Figure 8:**
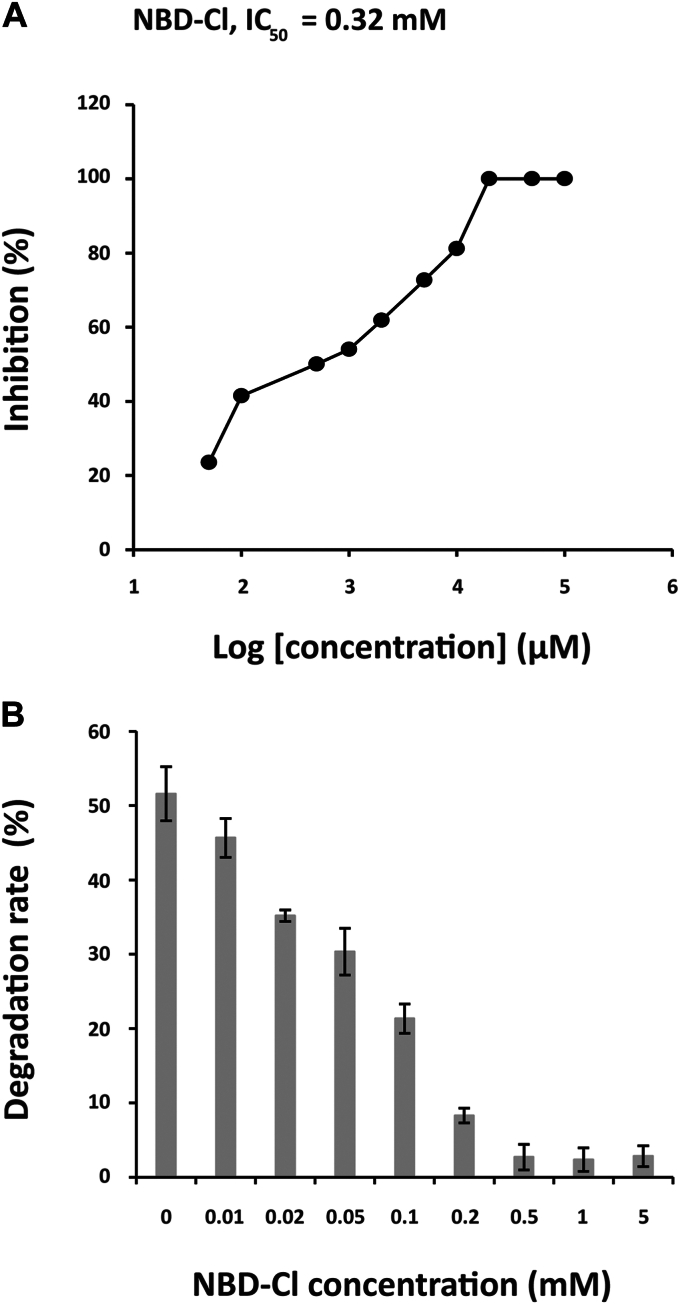
**Inhibitory activity of 4-chloro-7-nitro-2,1,3-benzoxadiazole against TCpGST- and papaya peel–catalyzed thiram (1) degradation.***A*, inhibitory curve of NBD-Cl against TCpGST. The reaction system contained 0.18 mM 1, 0.01 U TCpGST, and 0.05, 0.1, 0.5, 1, 2, 5, 10, 20, 50, and 100 mM NBD-Cl (total volume = 50 μl). The degradation rates were determined by HPLC after shaking at 200 rpm and 28 °C for 1 h. The control experiment was carried out in the absence of NBD-Cl. *B*, inhibitory activity of NBD-Cl against papaya peel–catalyzed degradation of 1. The reaction system consisted of papaya peel (50 pieces of 0.250 × 0.250 cm in size), 0.18 mM 1, and 0.01, 0.02, 0.05, 0.1, 0.2, 0.5, 1, and 5 mM NBD-Cl (total volume = 50 μl). The degradation rates were determined by HPLC after shaking at 200 rpm and 28 °C for 10 h. The control experiment was carried out in the absence of NBD-Cl. NBD-C1, 4-chloro-7-nitro-2,1,3-benzoxadiazole; TCpGST, tau class glutathione S-transferase from Carica papaya.

To understand the contribution of the GSTs expressed by papaya peel in the degrading ability of papaya peel toward 1, the inhibitory activity of NBD-Cl was evaluated. 7-Nitro-2,1,3-benzoxadiazole derivatives, such as NBD-Cl, have been reported to bind to the H- and G-sites of GSTs, showing GST selective inhibitory activity (19). As expected, NBD-Cl inhibited TCpGST-catalyzed degradation of 1, showing an IC_50_ of 0.32 mM (Fig. 8*A*). Interestingly, NBD-Cl at 0.5, 1, and 5 mM inhibited the degrading activity of papaya peel by 94.5% to 95.4%, and the percent of inhibition could not be enhanced when applying higher concentrations of inhibitor (Fig. 8*B*). It must be noted that NBD-Cl alone did not promote the degradation of 1. Given that NBD-Cl is a GST-selective inhibitor (19), the results indicate that the degradation of 1 is primarily catalyzed by the papaya-expressed GSTs. According to the obtained results, there must be other degradation pathways in papaya peel that account for approximately 5% of the total degradation activity.

### TCpGST and PCpGST can degrade various fungicides commonly used in papaya

The ability of the recombinant enzymes to degrade the fungicides commonly used for the control of fungal pathogens in papaya fruit, including azoxystrobin (10), chlorothalonil (11), difenoconazole (12), fluxapyroxad (13), pyraclostrobin (14), tebuconazole (15), thiabendazole (16), trifloxystrobin (17), and triflumizole (18), was screened (13).

**Figure 9:**
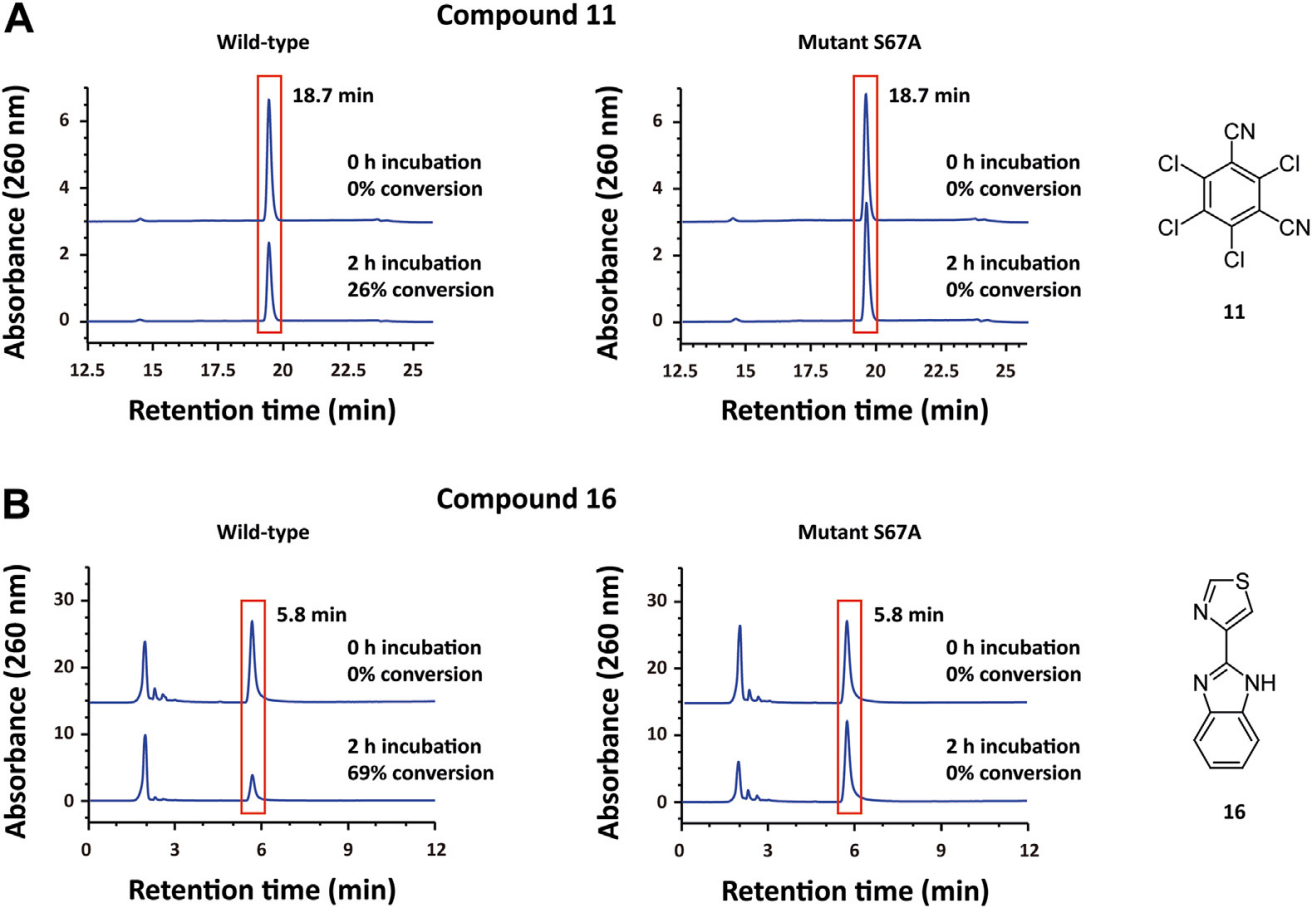
**Catalytic promiscuity of TCpGST against different fungicides commonly used in papaya.***A*, HPLC-based analysis of TCpGST-catalyzed degradation of chlorothalonil (11). The reaction system contained 0.2 mM 11 and 0.01 U TCpGST in 50 mM Tris buffer (total volume = 50 μl) and was shaken at 200 rpm and 28 °C. The reaction was stopped by adding 50 μl methanol. *B*, HPLC-based analysis of TCpGST-catalyzed degradation of thiabendazole (16). The reaction system contained 0.2 mM 16 and 0.01 U TCpGST in 50 mM Tris buffer (total volume = 50 μl) and was shaken at 200 rpm and 28 °C. The reaction was stopped by adding 50 μl methanol. The degradation of 11 and 16 was monitored using an HPLC system equipped with a C18 column at 260 nm. The mobile phase (1 ml/min) consisted of a gradient from 30% to 77% acetonitrile from 0 to 20 min. TCpGST did not catalyze the degradation of azoxystrobin (10), difenoconazole (12), fluxapyroxad (13), pyraclostrobin (14), tebuconazole (15), trifloxystrobin (17), and triflumizole (18). TCpGST, tau class glutathione *S*-transferase from *Carica papaya*.

LCpGST did not degrade any of the mentioned fungicides, suggesting again that lambda class GSTs do not show the ability to degrade organic substances. Interestingly, TCpGST catalyzed the degradation of 11 (Fig. 9*A*) and 16 (Fig. 9*B*), but this enzyme did not show any activity toward the rest of the fungicides. The degradation rates of 11 and 16 in the presence of TCpGST after 2 h were 26% and 69%, respectively. However, no obvious degradation was detected in the absence of TCpGST. The degradation of 11 and 16 was only observed when using the WT enzyme. However, mutant S67A did not show any degradation activity, demonstrating that the residue S67 shows a key influence in the degradation activity toward different organic compounds. Furthermore, PCpGST degraded 11 and 13 (Fig. S18). This result indicates that the substrate promiscuity of GSTs strongly varies depending on each specific enzyme.

The tau class GSTs from jujube was suspected to degrade 11 (38), which is consistent with the reported ability of TCpGST to degrade this compound. The fungicide 11 is a benzene-1,3-dicarbonitrile substituted by four chloro groups and can form adducts with GSH by the action of GSTs (59). 11 can be transformed into 4-hydroxychlorothalonil using microorganisms (60). 16 is a benzimidazole substituted in position 2 with a thiazole. 16 can be degraded by microorganisms to produce 5-hydroxythiabendazole (61). The fact that 11 and 16 can be hydroxylated to promote its degradation supports the key influence of the residue S67. However, further experiments are necessary to identify the degradation products and predict the reaction mechanism. Although Axarli *et al*. indicated that diphenyl ether fungicides showed the lowest energy conformation in the G-site of the tau class GST from *G. max* (31), the diphenyl ether fungicides 10 and 12 were not degraded by TCpGST. On the other hand, 13 is a carboxamide, which can be hydrolyzed in natural environments (62).

This study confirms for the first time the ability of plant GSTs to degrade fungicides. It must be noted that only the studied tau and phi classes GSTs showed fungicide degradation activity, and these two classes of GSTs can be only found in plants. The obtained results indicated that plant GSTs may have broad implications in the food industry to degrade fungicides, as well as other toxic residues before human consumption. Our results demonstrated that plant GSTs can be easily expressed in *E. coli* using standard procedures, suggesting that plant GSTs can be produced on a large scale without facing complex and expensive conditions. The induction of plant GST activity in postharvest fruits and vegetables may be also a suitable approach to enhance their intrinsic degradation ability. Currently, methods for the degradation of fungicides in food are lacking, highlighting the significance of this new approach. Further research is necessary to understand how to apply and optimize the degradation effects of plant GSTs in food products.

## Experimental procedures

### Gene cloning and construction of the expression vectors

Five hundred milligrams of papaya tissue were ground into powder with liquid nitrogen. Total RNA was extracted using TRIzol reagent (Ambion). The complementary DNA was synthesized using the HiScript III RT SuperMix for real-time PCR (+genomic DNA wiper) Kit (Vazyme). The oligonucleotide primers for amplifying the putative *TCpGST*, *PCpGST*, and *LCpGST* genes were 5′-ATGGCGGACGAGGTTGTT-3′ (forward primer)/5′-TCAAATCCCAAGAGCCTTCCTG-3′ (reverse primer), 5′-TAAGAAGGAGATATACATATGGCACCCGTCAAGGTT-3′ (forward primer)/5′-GAGTGCGGCCGCAAGCTTCATCTTTTGCAAAGCAATGACCTT-3′ (reverse primer), and 5′-TAAGAAGGAGATATACATATGGCTACTGGTGCGGAA-3′ (forward primer)/5′-GAGTGCGGCCGCAAGCTTAACCTCAATCCATGTTGCTAGTTT-3′ (reverse primer), respectively. After denaturation at 98 °C for 5 min, PCR amplification was performed using 30 PCR cycles that consisted of 30 s at 98 °C, 30 s at 59 °C (for *TCpGST*) or 30 s at 53 °C (for *PCpGST* and *LCpGST*), 1 min at 72 °C, and a final extension of 10 min at 72 °C (Table S7).

The PCR fragments were purified on an agarose gel, digested with the restriction endonucleases *Nde* I and *Hind* III, and subsequently ligated into a pET-30a expression vector in-frame with the hexa-histidine tag at the C terminus, which was predigested using the same restriction endonucleases. The ligation mixtures were transformed into *E. coli* DH5α competent cells and were selected on LB agar medium (5 g yeast extract, 10 g tryptone, 10 g sodium chloride, and 15 g agar in 1 L of distilled water; pH adjusted to 7–7.2) containing 50 μg/ml kanamycin. Transformants containing the expected plasmid construct were screened by Sanger DNA sequencing (GenScript). After extraction of the plasmid, the expression vector was transformed into *E. coli* BL21 (DE3) competent cells. Nucleotide and amino acid sequences of TCpGST, PCpGST, and LCpGST are shown in Table S8 (GenBank accession numbers: OQ870530, PP125178, and PP125179).

### Expression and purification

A single *E. coli* BL21 (DE3) colony was transferred into 5 ml LB medium containing 50 μg/ml kanamycin, and the culture was shaken at 37 °C for 16 h. The cells were then collected, resuspended in 400 ml LB medium, and shaken at 200 rpm and 37 °C until the *A*_600_ value reached 0.5. Recombinant protein expression was induced by adding 1 mM IPTG (400 μl) to the fermentation broth. After 4 h of shaking at 25 °C, cells were harvested by centrifugation at 5000*g* for 20 min, resuspended in lysis buffer (50 mM Tris, 100 mM NaCl, and 1% (*v*/*v*) Triton X-100; pH adjusted to 7.4), and disrupted by sonication (40 ON/OFF cycles with 20 μm probe amplitude for 15 s) using an ultrasonic homogenizer (JY92-IN, Ningbo Scientz Biotechnology).

The cell lysate supernatant was collected by centrifugation at 10,000*g* for 30 min and loaded onto a Ni-NTA affinity column (Wuxi Tianyan). After washing the column with 50 ml of washing buffer (50 mM Tris/HCl; pH adjusted to 7.2), the recombinant enzymes were then eluted with elution buffer (50 mM Tris/HCl and 300 mM imidazole; pH adjusted to 7.4). Protein expression and purification were monitored by SDS-PAGE using Coomassie brilliant blue G-250. Elution fractions showing the highest purity were pooled and stored in 20% glycerol (*v*/*v*) at −80 °C. The protein concentration was measured using a Bradford Protein Quantification Kit (Sangon Biotech).

### Enzymatic degradation assay

The enzymatic activities of TCpGST, PCpGST, and LCpGST were evaluated using 1, 5, 6, 7, 8, 10, 11, 12, 13, 14, 15, 16, 17, and 18 as substrates. The reaction system contained 0.2 mM fungicide in 50 mM Tris buffer (45 μl; pH 7.2) and recombinant enzyme (5 μl; 0.01 U). One enzyme unit was defined as the amount of enzyme that catalyzes the conversion of 1 μmol of GSH per minute. The control group consisted of 0.2 mM fungicide in 50 mM Tris buffer (50 μl; pH 7.2), in the absence of enzymes. After 2 h at 200 rpm and 28 °C, 50 μl methanol was added to stop the reaction. Five repetitions were carried out for each treatment condition.

The degradation of the fungicides was monitored using an HPLC system (Agilent 1200 Series) equipped with a C18 column (End-Capped Reverse-Phase Eclipse XDB-C18 column, 250 × 4.6 mm, Agilent) and UV detector. The injection volume was 10 μl, and the running temperature was 25 °C. The detection was conducted at 260 nm at a flow rate of 1 ml/min. The mobile phase consisted of A: ultrapure water and B: acetonitrile. The elution gradient was as follows: 30% to 77% B (from 0 to 20 min) and 30% B (from 20 to 30 min). 1, 5, 6, 7, 8, 10, 11, 12, 13, 14, 15, 16, 17, and 18 appeared a 12.5, 12.4, 14.2, 14.3, 14.1, 16.3, 18.7, 20.3, 16.5, 21.7, 17.0, 5.8, 15.1, and 21.7 min retention time, respectively, using the mentioned HPLC conditions. Calibration curves were established using commercial compounds. The concentration of the compounds was calculated according to the peak area.

### Structure analysis of TCpGST

The three-dimensional structure of TCpGST was predicted using Phyre2 according to the reported X-ray three-dimensional structure of the tau class GST from *G. max* (https://www.ebi.ac.uk/pdbe/entry/pdb/2vo4) (28). To identify the sites where 1 and 8 were located, the docked conformation of TCpGST in complex with the substrates was generated using the CB-Dock software (https://cadd.labshare.cn/cb-dock/php/blinddock.php) (63). The interactions and distances between the substrates and residues of the TCpGST active site were calculated using SwissDock (Table S9) (64).

### Site-directed mutagenesis

TCpGST G-site residues F10, S13, K53, I54, E66, and S67, which were close to 1 according to the docking study, were chosen for site-directed mutagenesis. SnapGene software (version 6.1; https://www.snapgene.com/updates/snapgene-6-1-0-release-notes) was used to design site-specific mutation primers (Table S10). All residues were mutated to alanine. The PCR amplification was performed using the pET-30a expression vector containing the *TCpGST* gene as the template with 25 PCR cycles of 10 s at 98 °C, 15 s at 54 to 59 °C, and 30 s at 72 °C and a final extension of 10 min at 72 °C (Table S11). Green buffer (5.1 μl) and *Dpn* Ⅰ (1 μl; Takara) were directly added to the PCR products, and the resulting solution was incubated at 37 °C for 3 h to remove the template DNA. *Exnase* ⅠⅠ (Vazyme) was then added to the digested product to induce the homologous recombination reaction, and the resulting solution was incubated at 37 °C for 30 min. Selection of transformants and mutant proteins expression and purification were carried out as described in the “Expression and purification” section.

### HPLC- and NMR-based analyses of dimethyl dithiocarbamate (2) and dimethyl dithiocarbamoylsulfenic acid (9)

The reaction was scaled up to 1 ml (following the conditions described in the “Enzymatic degradation assay” section) and contained 43.3 μg (0.18 μmol) of 1. The reaction was shaken at 200 rpm and 28 °C for 24 h. LC-MS analyses were carried out using a Triple Quadrupole/AB SCIEX 5500 QTRAP system (Sciex). After stopping the reaction following the conditions described in the “Enzymatic degradation assay” section, the compounds were directly detected in the solution using an LC-MS instrument (Agilent 1290-Sciex Qtrap 5500) with an Agilent SB-C18 column (21 × 75 mm) at 25 °C. The conditions reported by Gupta *et al*. were followed for the detection (44). The mobile phase consisted of 100% methanol at a constant flow rate of 1 ml/min for 15 min (injection volume: 10 μl). More details about the MS detection conditions are given in Table S12. To examine the degradation of 2 and 9, the reaction was stopped by adding 1 ml methanol, and the mixture was incubated at 28 °C for 0, 0.5, 1, and 2 h. Standard compound 2 (Macklin) was used to establish a linear calibration, while compound 9 was purified from the reaction medium and used to establish a linear calibration.

Compounds 2 (9 μg, 0.075 μmol, 41.7% yield) and 9 (11 μg, 0.080 μmol, 44.5% yield) were purified from the reaction medium by silica chromatography (20 g silica) using methanol as the mobile phase (2, R_f_ = 0.5; 9, R_f_ = 0.8). The identity of the compounds was confirmed by MS (Agilent 1290-Sciex Qtrap 5500) and ^1^H and ^13^C NMR (Bruker AVANCE III HD 400M). Only the signals corresponding to the expected compounds were detected in the NMR spectra, demonstrating that the compounds were obtained with high purity.

### GC-MS–based analysis of carbon disulfide (4)

To confirm the presence of 4, the enzymatic degradation of 1 was carried out as indicated in the “Enzymatic degradation assay” section for 0, 0.5, 1, 3, 5, and 10 h using a 20 ml sealed glass tube (500 μl reaction volume). After stopping the reaction with methanol (500 μl), the solution was kept at 28 °C for 2 h, and 1 ml of the headspace gases was injected in a GC-MS instrument (Thermo Fisher Scientific). More details about the MS detection conditions are shown in Table S13. Standard compound 4 (Macklin) was used to establish a linear calibration. 4 appeared at 1.91 min retention time in the chromatogram. The degradation of 1 was simultaneously monitored using the HPLC method described in the “Enzymatic degradation assay” section.

### Measurement of GST activity in papaya

Papaya fruit was immersed in an aqueous solution (400 ml) containing 10 mM 1 for 20 min. The control experiment was carried out in the absence of 1. After the treatment, the papaya was stored at 28 °C and 75% relative humidity. Papaya tissue (0.01 g), containing either peel or mesocarp, was collected with a sterilized knife after 0, 1, 3, and 5 days. GST activity was measured following the procedure described by Jo *et al*. (33). The enzymatic activity was evaluated using the changes in absorbance at 340 nm produced during the formation of 2,4-dinitrophenyl-glutathione from 1-chloro-2,4-dinitrobenzene and GSH. The content of GST was expressed as units per gram of papaya tissue. Five repetitions were carried out for each treatment condition and time point.

### Thiram-degrading ability of papaya peel

Papaya peel tissue (1.25 × 1.25 cm) was extracted using a sterilized knife, sectioned into 0.25 × 0.25 cm pieces, and added to an Eppendorf tube containing 0.18 mM 1 (500 μl). 1 was detected in the reaction medium by HPLC using the conditions described in the “Enzymatic degradation assay” section, while the presence of 4 and 9 were analyzed following the conditions indicated in the previous sections.

### Assay of NBD-Cl inhibitory activity

First, the inhibitory effect of NBD-Cl on the activity of recombinant TCpGST was analyzed. The reaction system consisted of recombinant TCpGST (5 μl; 0.01 U), 2 μl 1 (0.18 mM final concentration), 2 μl inhibitor (0.05, 0.1, 0.5, 1, 2, 5, 10, 20, 50, and 100 mM final concentration), and 46 μl H_2_O. The control experiment was carried out in the absence of NBD-Cl. The degradation rates were determined after shaking at 200 rpm and 28 °C for 1 h. 1 was detected by HPLC following the method described in the “Enzymatic degradation assay” section.

Furthermore, the inhibitory effect of NBD-Cl on the papaya peel degrading activity was analyzed. In this case, the reaction system consisted of papaya peel (sectioned into 0.25 × 0.25 cm pieces), 2 μl 1 (0.18 mM final concentration), 2 μl inhibitor (0.01, 0.02, 0.05, 0.1, 0.2, 0.5, 1, and 5 mM final concentration), and 46 μl H_2_O. The control experiment was carried out in the absence of inhibitors. The degradation rates were determined by HPLC after 10 h at 200 rpm and 28 °C. Five replicates were carried out for each treatment condition and time point.

### Statistical analysis

Five replicates were performed for each experiment, except for the real-time quantitative reverse transcription PCR analysis, which consisted of three replicates. Statistical analyses were performed using Statistical Package, version 20.0. GST activity and real-time quantitative reverse transcription PCR data were processed using Tukey’s test in one-way ANOVA *via* post hoc multiple comparisons (*p* ≤ 0.05).

## Data availability

Genetic material used in this research will be made available on request (contact, pedro@ntu.edu.cn). Nucleotide and amino acid sequences were submitted to GenBank (GenBank accession number: OQ870530, PP125178, and PP125179). All remaining data are contained within the published article.

## Supporting information

This article contains Supporting information (23, 33, 65, 66).

## Conflict of interest

The authors declare that they have no conflicts of interest with the contents of this article.
