# Supplementary material for: G-site residue S67 is involved in the fungicide-degrading activity of a tau class glutathione S-transferase from Carica papaya
Source: J Biol Chem. 2024 Feb 28;300(4):107123. doi: 10.1016/j.jbc.2024.107123 (PMC10958117; doi:10.1016/j.jbc.2024.107123)
Supplement: Supporting Figures S1–S18 and Tables S1–S14 [file mmc1.pdf]

Supporting Information

G-site residue S67 is involved in the fungicide-degrading activity of a tau class glutathione *S*-transferase from *Carica papaya*

Su-Yan Wang[1,‡], Yan-Xia Wang[1,‡], Sheng-Shuo Yue[1], Xin-Chi Shi[1], Feng-Yi Lu[1], Si-Qi Wu[1], Daniela D. Herrera-Balandrano[1,*], and Pedro Laborda[1,*]

[1]School of Life Sciences, Nantong University, Nantong, China.

[‡]These authors equally contributed to this work.

[*] For correspondence: Pedro Laborda, pedro@ntu.edu.cn; Daniela D. Herrera-Balandrano, daniela.herrera@ntu.edu.cn.

1. Supplementary methods

General information

Red-flesh 'RB1' papayas were used in the experiments. The visual maturity of the papayas was determined on the scale proposed by Basulto et al. (1), where yellow skin corresponded to mature stage 4. Buffers, salt, and other chemicals used in this study were purchased at the highest grade from various commercial suppliers.

DNA polymerases (PrimeSTAR Max Premix (2X)), restriction endonucleases, T4 ligases, and DNA Gel Purification Kit were purchased from Takara. Plasmid Extraction Kit was from Solarbio. Oligonucleotide primers were purchased from GenScript.

Construction of the phylogenetic tree (Fig. S4)

A phylogenetic tree based on the amino acid sequences was constructed with TCpGST and reference strains retrieved from UNIPROT. The phylogenetic tree was constructed using MEGA11 with the JTT matrix model (2). The percentage of trees in which the associated taxa clustered together is shown next to the branches. Pairwise distances were estimated using the Maximum Composite Likelihood (MCL) approach, and then selecting the topology with a superior log likelihood value (–1388.85). There were a total of 48 positions in the final dataset. The gamma distribution (5 categories, +*G*) parameter was 4.8278, and the number of bootstrap replications was 1000.

Calculation of kinetic parameters

Kinetic parameters were determined using **1** and **8**. The reaction system consisted of 4 μL substrate (at 0.006, 0.008, 0.01, 0.02, 0.04, 0.05, 0.08, 0.1, 0.15, and 0.18 mM final concentration), recombinant enzyme (10 μL; 0.02 U), and 86 μL H_2O. After 2 h, the reaction was stopped by adding 100 μL methanol. The degradation was monitored by HPLC. V_{max}, K_m, and k_{cat} values were calculated by applying a non-linear regression model using the OriginPro 9.1 data analysis software.

Biochemical characterization of **TCpGST**

Optima temperature and pH of TCpGST, as well as the effects of metals and additives on TCpGST activity, were determined. To evaluate temperature optimum, reaction mixtures were incubated at temperatures ranging from 4 to 60 ºC. pH optimum was investigated from pH 3.0 to pH 10.0. Negative controls were prepared in the absence of TCpGST. To evaluate the effect of metal ions on TCpGST activity, the enzymatic reactions were performed in the presence of 1 mM Co (II), Cu (II), Fe (II), Fe (III), and Zn (II). All metals were added to the reaction using the chloride forms. The metal dependency of TCpGST was evaluated by adding 1 mM EDTA in the reaction medium. The effect of denaturants and detergents on TCpGST activity was assessed using 2-mercaptoethanol (10 mM, 50 mM, and 100 mM), urea (0.5 M, 1 M, and 2 M), Triton X-100 (0.1%, 0.5%, and 1% *v/v*), and SDS (0.1%, 0.5%, and 1% *v/v*).

Activity assay of mutant enzymes towards thiram **(Fig. 3*C*)**

The activity tests of the mutant proteins towards **1** were performed as indicated in

the “Enzymatic degradation assay” section. The positive control was carried out using wild-type TCpGST, while the negative control experiment was carried out in the absence of enzymes.

Activity assay of mutant enzymes towards glutathione

The assay reported by Jo et al. was followed to measure the enzymatic activity towards glutathione (3). The reaction system contained 10 mM glutathione (50 μL), 10 mM 1,2-dichloro-4-nitrobenzene (50 μL), 15 mg/mL enzyme (50 μL), and 50 mM Tris/HCl buffer (350 μL; pH 7.2). The reaction solution was incubated at 37 ºC for 5 min. The enzymatic activity was evaluated using the changes in absorbance at 340 nm produced during the formation of 2,4-dinitrophenyl-glutathione from 1-chloro-2,4-dinitrobenzene and glutathione. The positive control was carried out using wild-type TCpGST, while the negative control experiment was carried out in the absence of enzymes. Five repetitions were carried out.

Expression level of TCpGST in papaya peel

Papaya fruit was treated with **1** as indicated in the previous section. The expression levels were examined at 0, 1, 3, and 5 days post-treatment. After grinding to powder the papaya peel tissue using liquid nitrogen, mRNA was extracted, and cDNA was synthesized using the procedure described in the “Gene cloning and construction of the expression vector” section. Quantitative real-time PCR (qRT-PCR) was performed using a set of two PCR primers with SYBR Green I Real Time PCR (Solarbio). The

PCR analysis was carried out using a 7500 Real Time PCR system (Applied Biosystems). Primers were designed using Primer Premier 5.0 (Premier Biosoft International) and are shown in Supporting Information Table S14. *Actin* was used as the reference gene, and the relative gene expression was calculated by the $2^{-\Delta\Delta CT}$ method. Three replicates were performed.

2. Supplementary figures

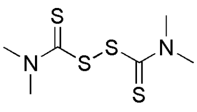

1

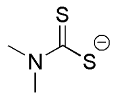

2

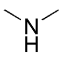

3

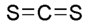

4

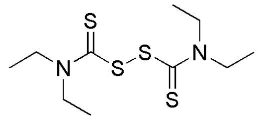

5

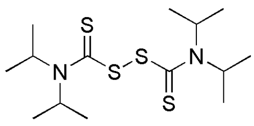

6

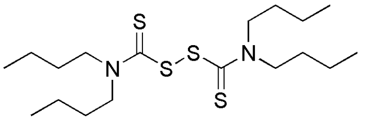

7

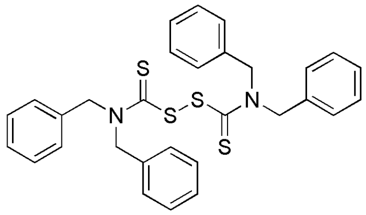

8

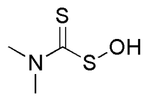

9

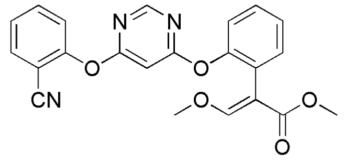

10

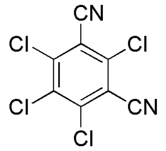

11

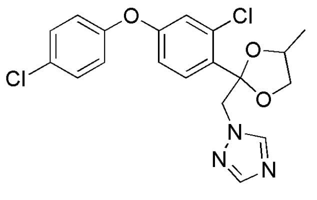

12

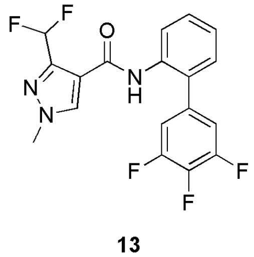

13

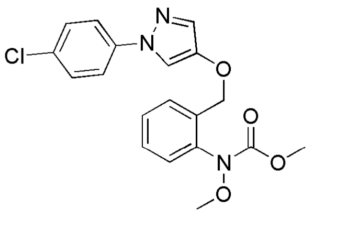

14

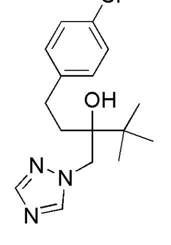

15

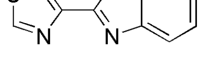

16

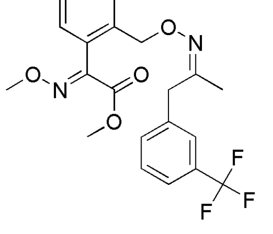

17

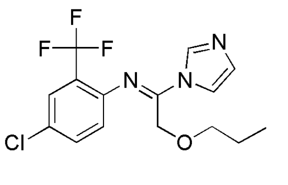

18

Fig. S1. Chemical structures of thiram (**1**), dimethyl dithiocarbamate (**2**), triethylamine (**3**), carbon disulfide (**4**), tetraethylthiuram disulfide (**5**), tetraisopropylthiuram disulfide (**6**), tetrabutylthiuram disulfide (**7**), tetrabenzylthiuram disulfide (**8**), dimethyl

dithiocarbamoylsulfenic acid (**9**), azoxystrobin (**10**), chlorothalonil (**11**), difenoconazole (**12**), fluxapyroxad (**13**), pyraclostrobin (**14**), tebuconazole (**15**), thiabendazole (**16**), trifloxystrobin (**17**), and triflumizole (**18**).

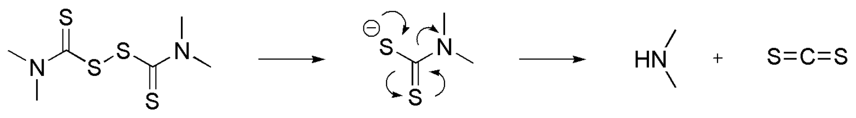

1 **2** **3** **4**

Fig. S2. Scheme showing the degradation of thiram (**1**) in aqueous solution. **1** degrades to dimethyl dithiocarbamate (**2**), which is subsequently transformed into triethylamine (**3**) and carbon disulfide (**4**).

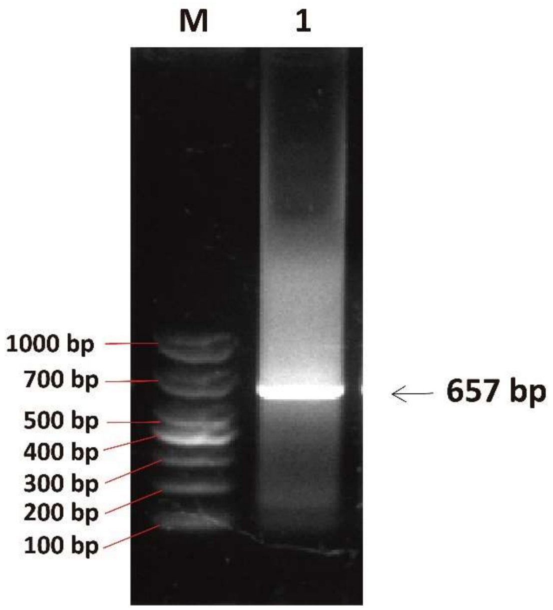

Fig. S3. Agarose gel electrophoresis of the amplified DNA segment encoding *TCpGST*.

M – DNA marker; **1** – *TCpGST*.

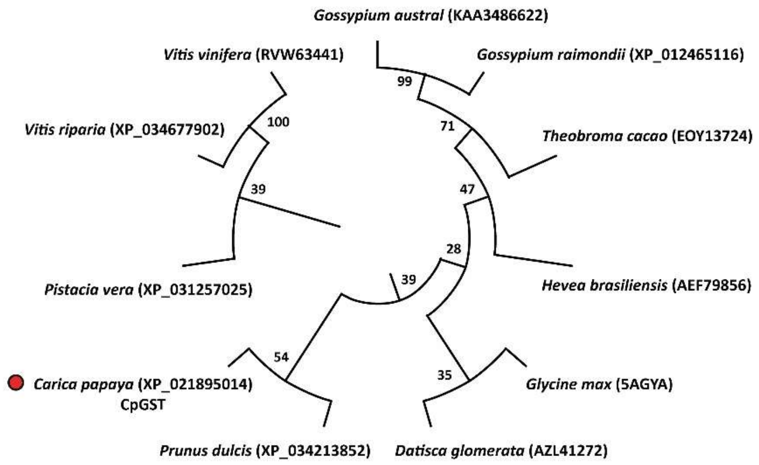

Fig. S4. Tree of TCpGST with some representative plant tau class GSTs based on the amino acids sequence. The phylogenetic tree was constructed using MEGA 11 with the JTT matrix model. Pairwise distances were estimated using the Maximum Composite Likelihood (MCL) approach, and then selecting the topology with a superior log likelihood value (–1388.85).

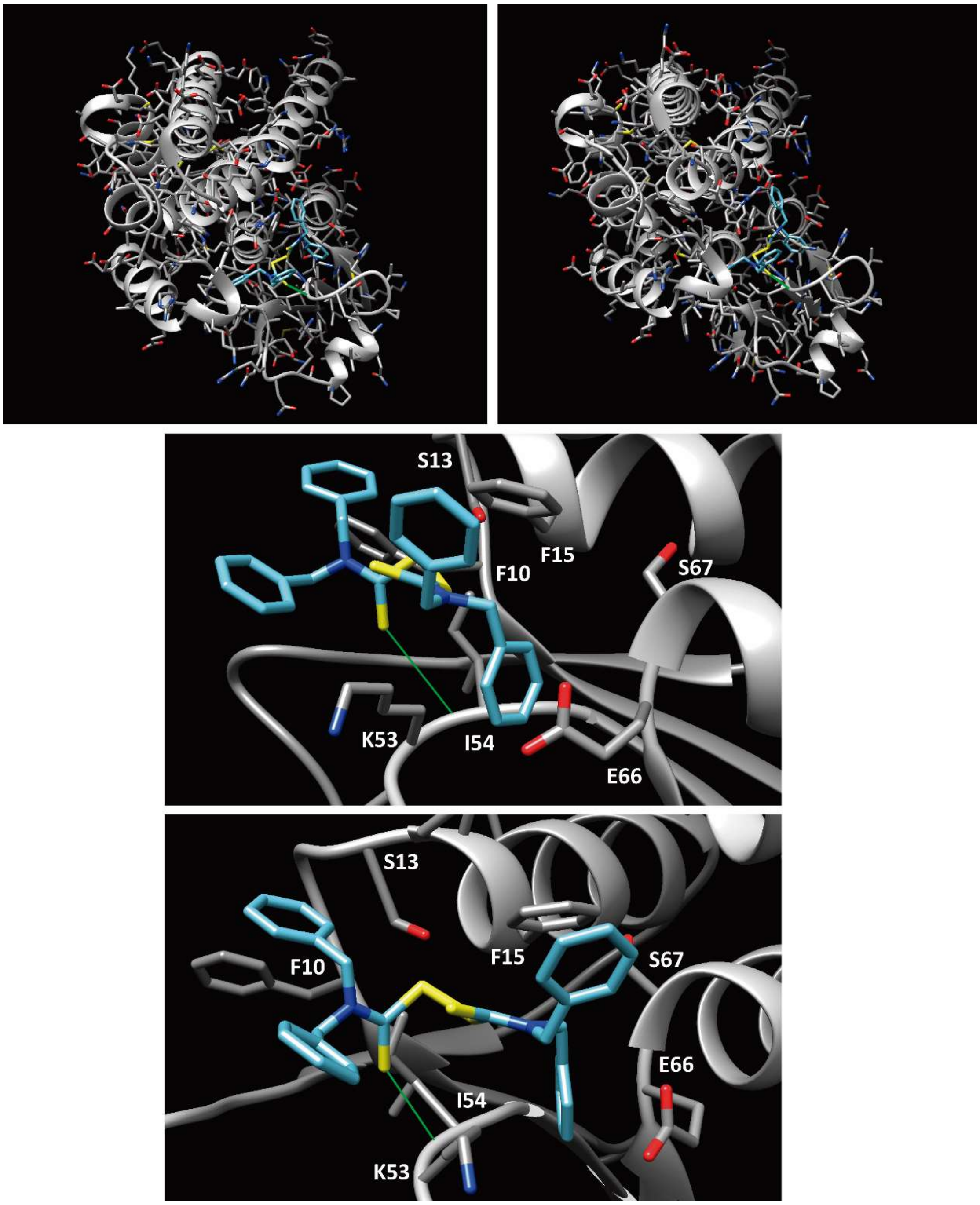

Fig. S5. Docked conformation of tetrabenzylthiuram disulfide (**8**) in the G-site of CpGST. Compound **8** showed high proximity to residues F10, S13, F15, K53, I54, E66 and S67.

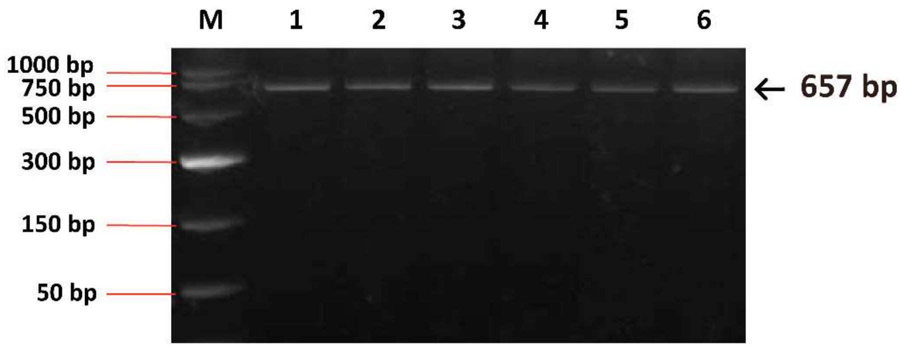

Fig. S6. Agarose gel electrophoresis of the amplified DNA segments encoding the mutant enzymes. **M**: DNA marker; **1**: F10A; **2**: S13A; **3**: K53A; **4**: I54A; **5**: E66A; **6**: S67A.

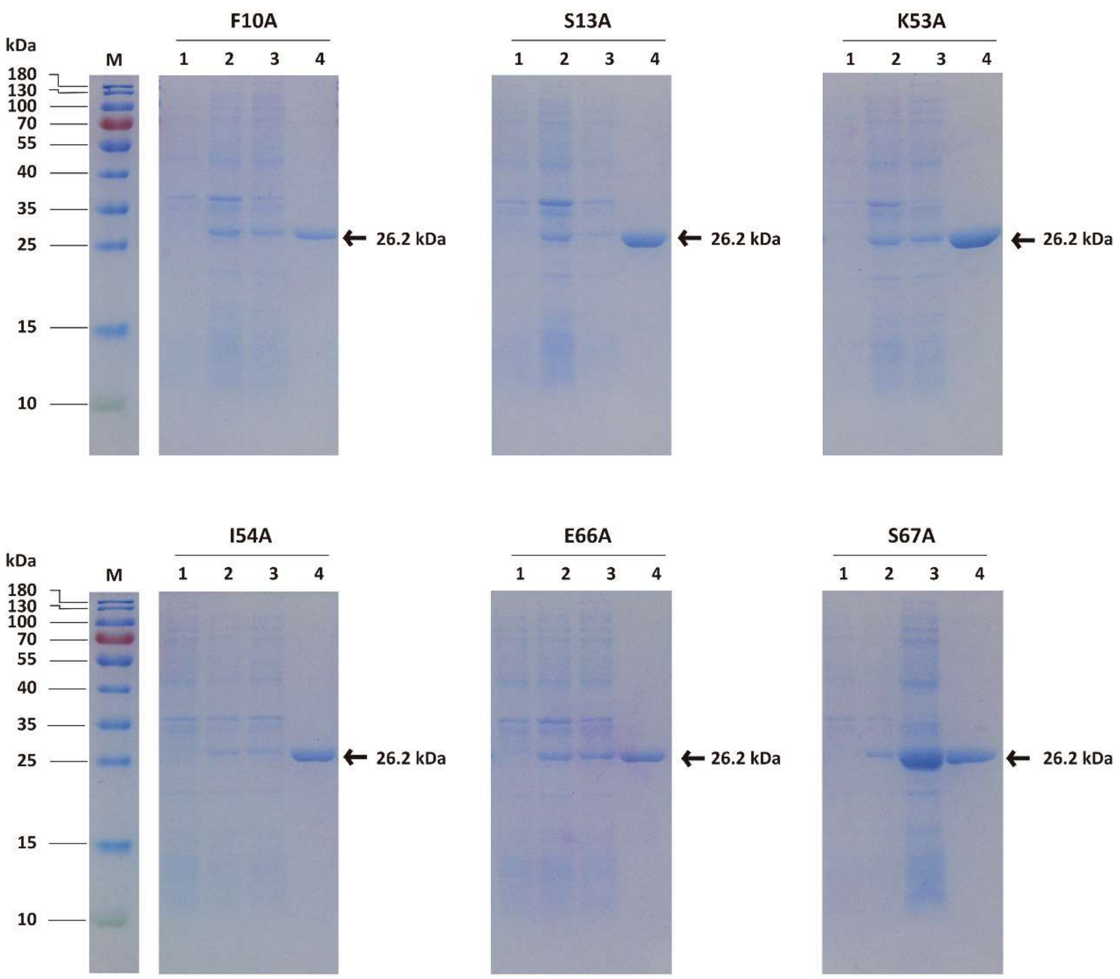

Fig. S7. SDS-PAGE analysis of recombinant mutant enzymes containing an additional *C*-terminal hexa-histidine tag. **M**: Protein marker; **1**: Cell pellets before induction; **2**: Cell pellets after induction; **3**: Supernatant of cell lysate; **4**: Ni-NTA purified enzyme.

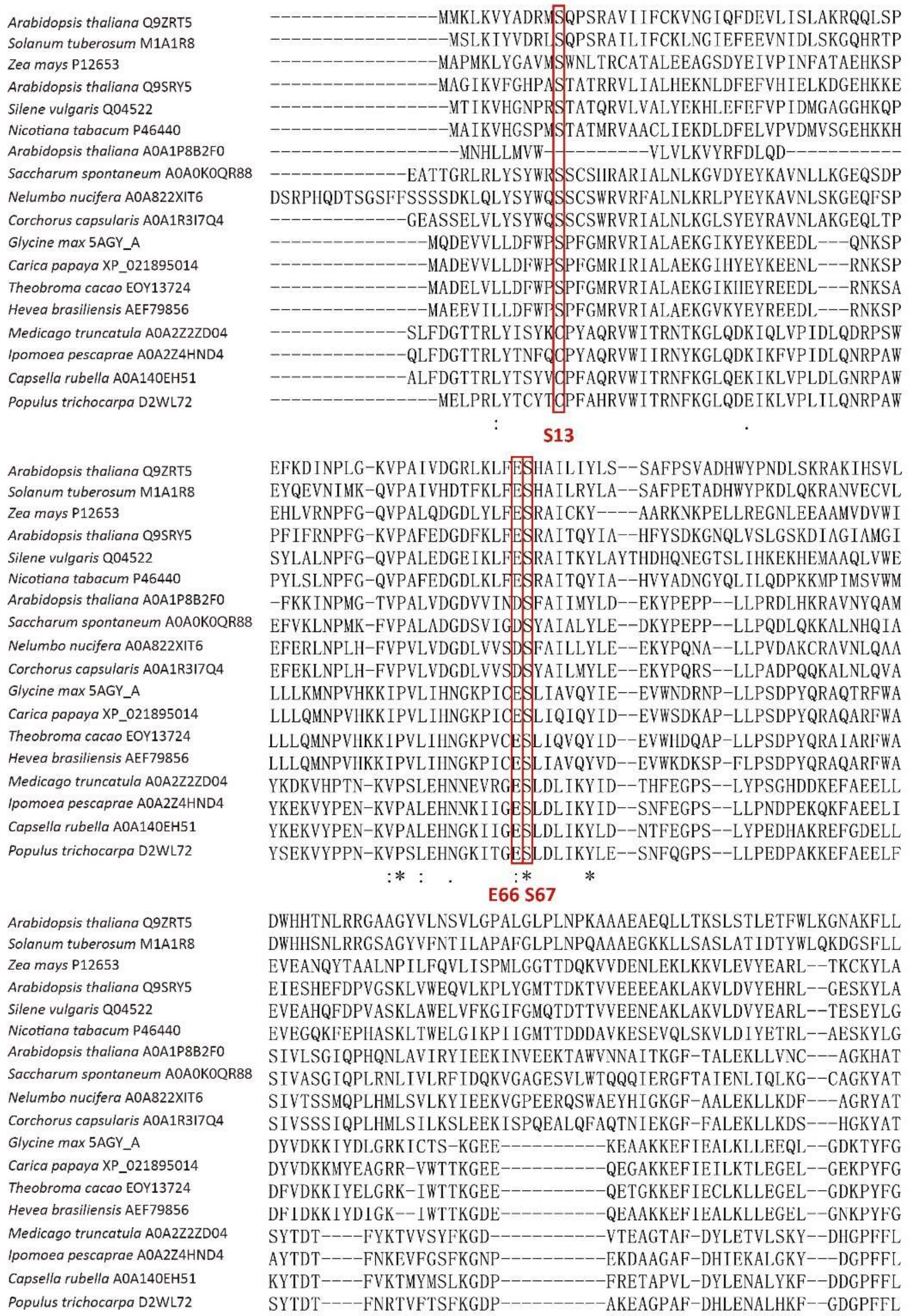

Fig. S8. Amino acid sequence alignment of CpGST and some representative GSTs from phi, zeta, tau, theta, and lambda classes.

Phi class GSTs from *Arabidopsis thaliana* (Q9SRY5), *Nicotiana tabacum* (P46440), *Silene vulgaris* (Q04522), and *Zea mays* (P12653);

Zeta class GSTs from *Arabidopsis thaliana* (A0A1P8B2F0), *Corchorus capsularis* (A0A1R3I7Q4), *Nelumbo nucifera* (A0A822XIT6), and *Saccharum spontaneum* (A0A0K0QR88);

Tau class GSTs from *Carica papaya* (XP_021895014), *Glycine max* (5AGY_A),

Hevea brasiliensis (AEF79856), and *Theobroma cacao* (EOY13724);

Theta class GSTs from *Arabidopsis thaliana* (Q9ZRT5), and *Solanum tuberosum* (M1A1R8);

Lambda class GSTs from *Capsella rubella* (A0A140EH51), *Ipomoea pescaprae* (A0A2Z4HND4), *Medicago truncatula* (A0A2Z2ZD04), and *Populus trichocarpa* (D2WL72).

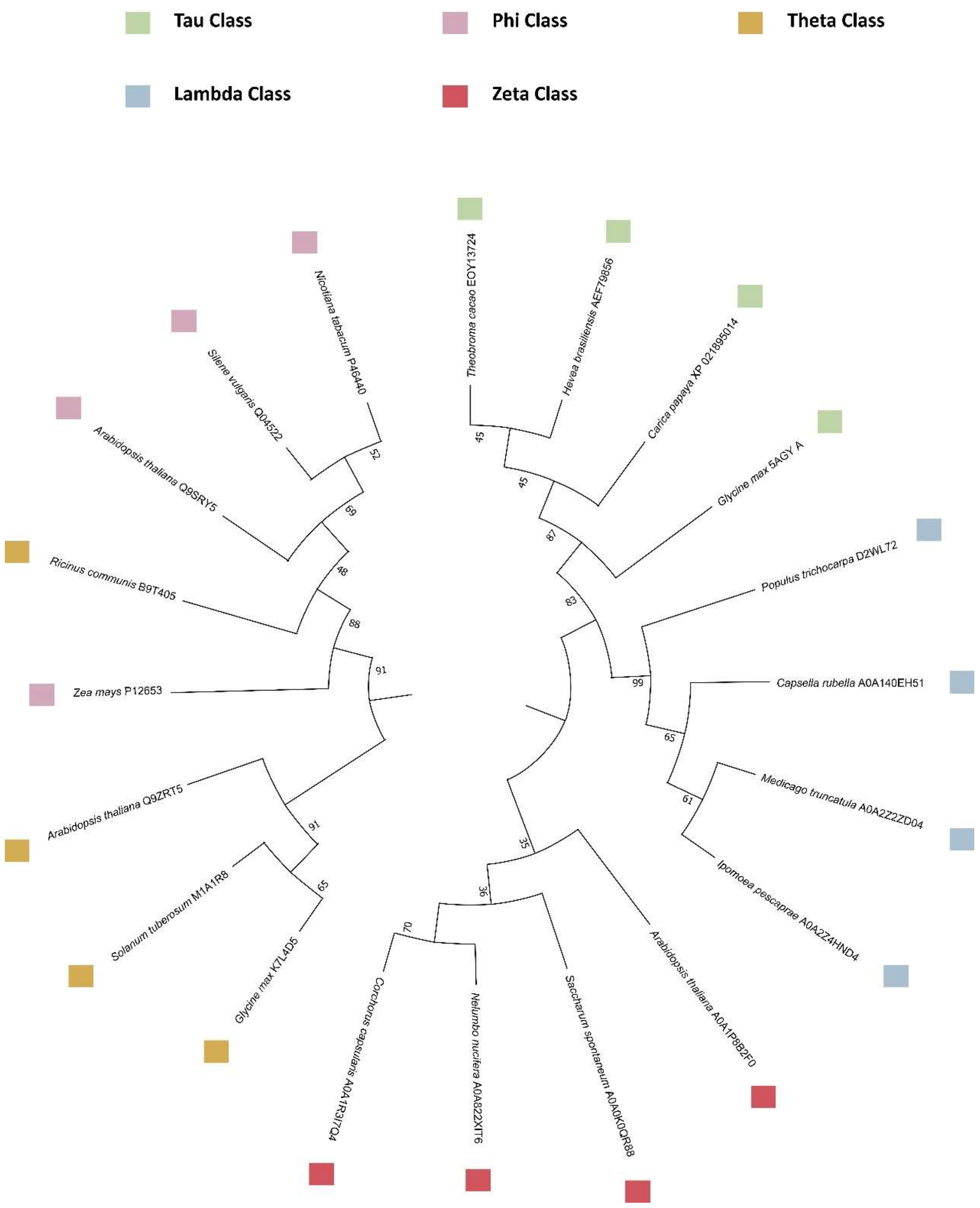

Fig. S9. Phylogenetic tree of CpGST with some representative plant GSTs based on the amino acid sequences.

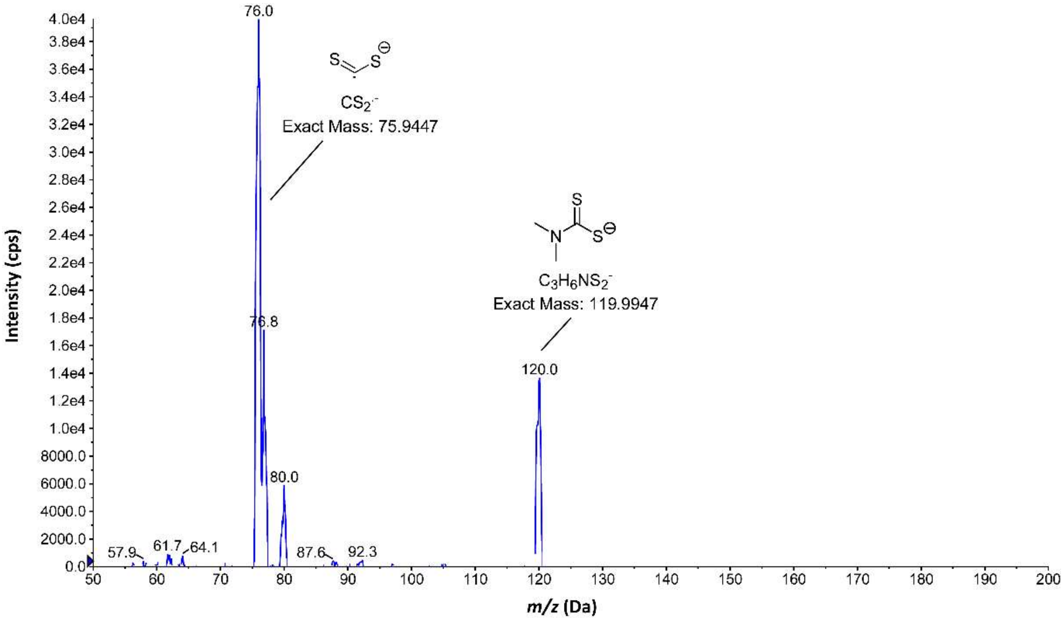

Fig. S10. MS/MS analysis of dimethyl dithiocarbamate (**2**) (*m/z* peak at 120.0 Da).

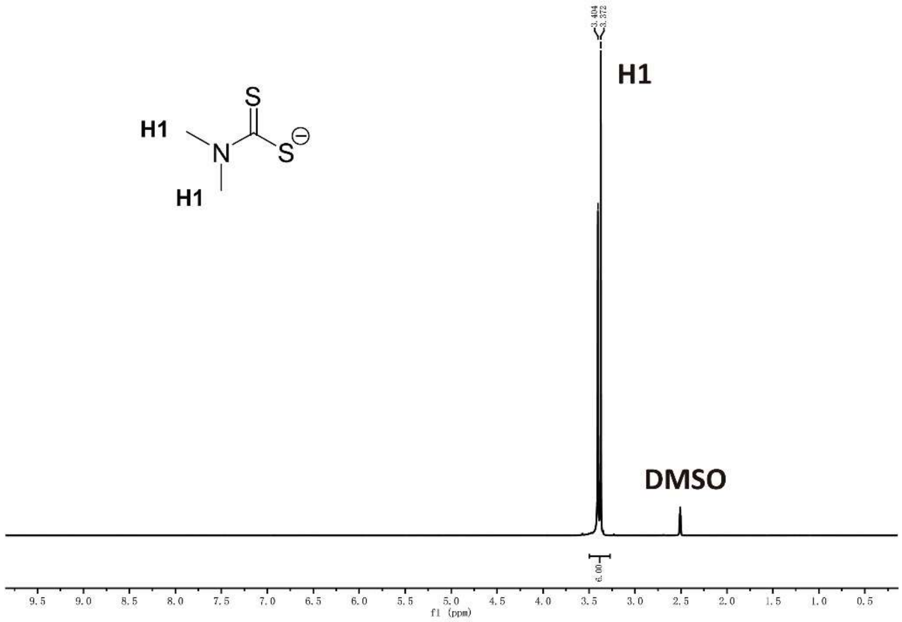

Fig. S11. ^{1}H NMR spectrum of purified dimethyl dithiocarbamate (**2**). The spectrum was registered on a Bruker AV-400 instrument using DMSO-d_6 as the solvent. The ^{1}H NMR spectrum showed two singlets at 3.40 and 3.37 ppm, corresponding to the rotamers of the methyl groups. No other signal was observed in the spectrum, suggesting that the dithiocarbamate is present in the anionic form.

^{1}H NMR (400 MHz, DMSO-d_6): δ 3.40, 3.37* (s, 6H, CH_3). (*) The two signals correspond to rotamers.

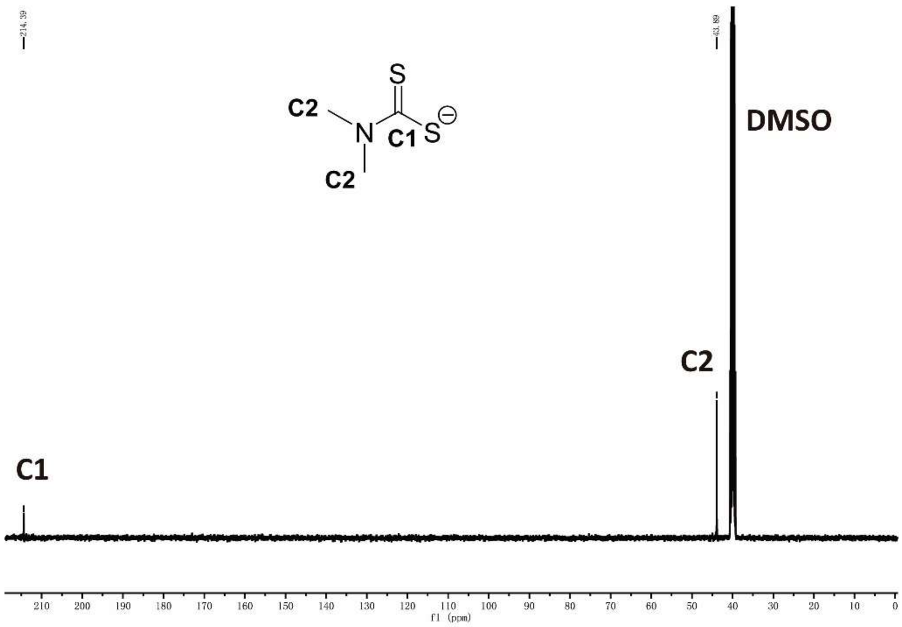

Fig. S12. ^{13}C NMR spectrum of purified dimethyl dithiocarbamate (**2**). The spectrum was registered on a Bruker AV-400 instrument using DMSO-d_6 as the solvent. The ^{13}C NMR spectrum showed signals at 214.39 (C=S) and 43.89 (CH_3) ppm. The signals corresponding to the methyl groups appears as only one singlet.

^{13}C NMR (125 MHz, DMSO-d_6): δ 214.39 (C=S), 43.89 (CH_3).

A

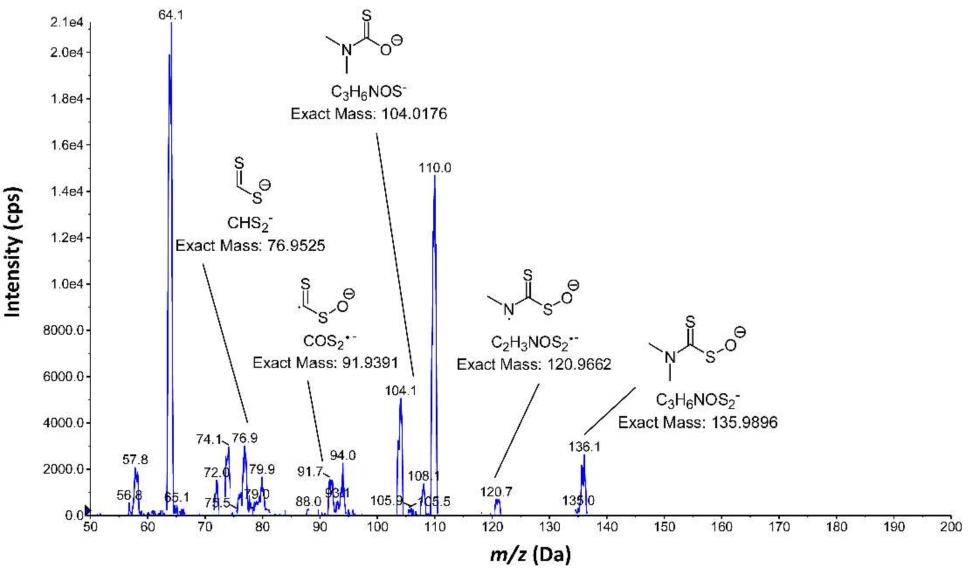

B

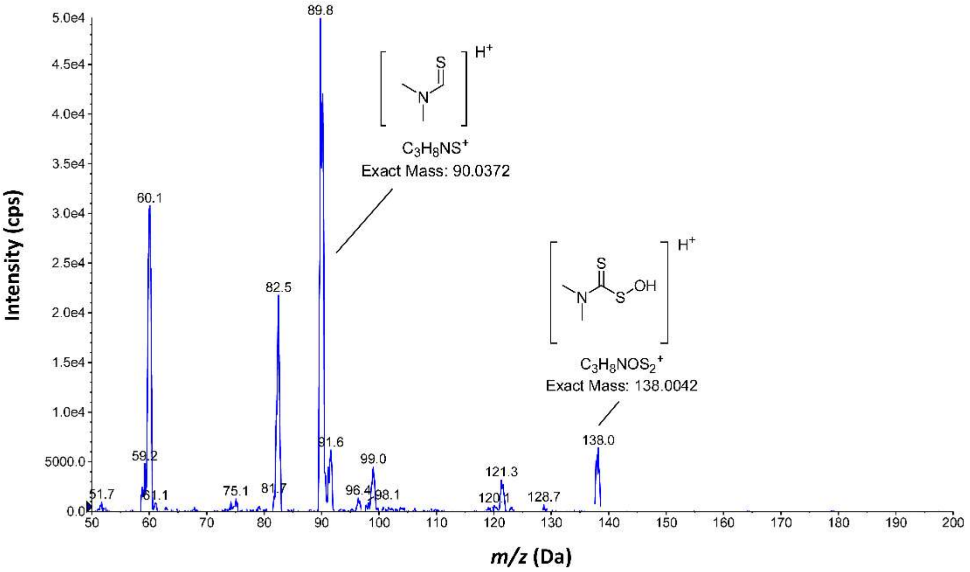

Fig. S13. MS/MS analysis of dimethyl dithiocarbamoylsulfenic acid (**9**). *A*, Negative mode (*m/z* peak at 136.1 Da). *B*, Positive mode (*m/z* peak at 138.0 Da).

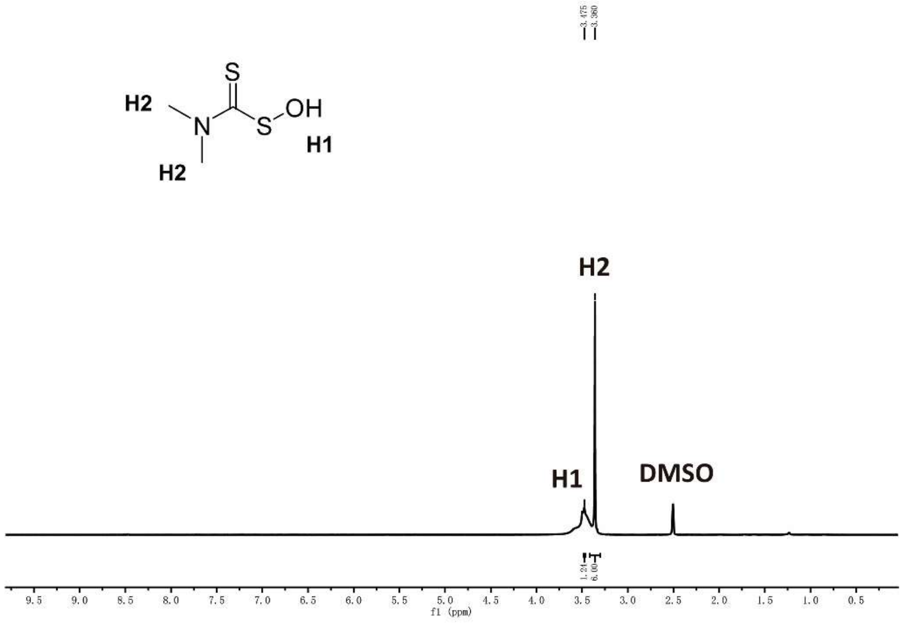

Fig. S14. ^{1}H NMR spectrum of purified dimethyl dithiocarbamoylsulfenic acid (**9**). The spectrum was registered on a Bruker AV-400 instrument using DMSO-d_6 as the solvent. The hydroxy group appeared as a broad singlet at 3.48 ppm. The methyl groups appeared as only one singlet at 3.36 ppm, indicating the presence of only one rotamer.

^{1}H NMR (400 MHz, DMSO-d_6): δ 3.48 (broad s, 1H, OH), 3.36 (s, 6H, CH_3).

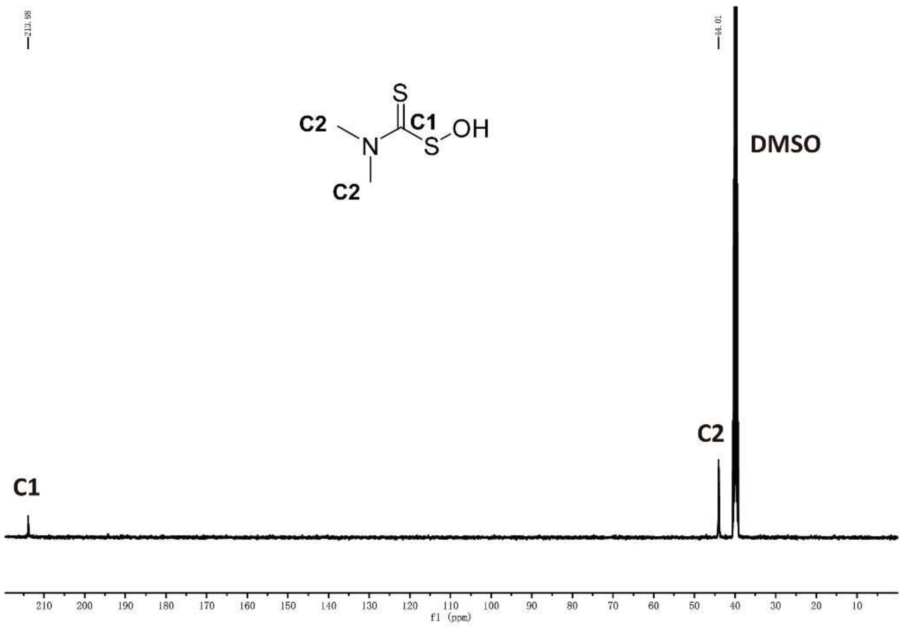

Fig. S15. ^{13}C NMR spectrum of purified dimethyl dithiocarbamoylsulfenic acid (**9**). The spectrum was registered on a Bruker AV-400 instrument using DMSO-d_6 as the solvent.

^{13}C NMR (125 MHz, DMSO-d_6): δ 213.88 (C=S), 44.01 (CH_3).

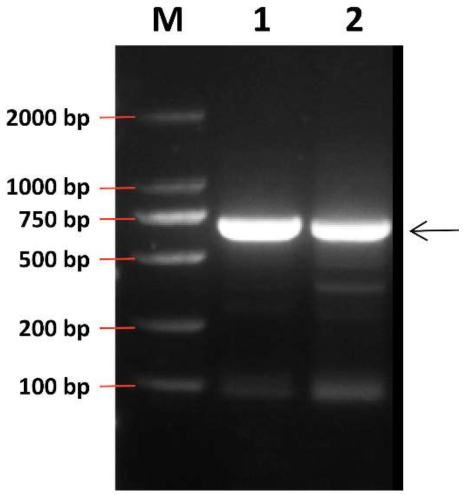

Fig. S16. Agarose gel electrophoresis of the amplified DNA segments encoding PCpGST and LCpGST. **M** – DNA marker; **1** – *PCpGST* (639 bp); **2** – *LCpGST* (618 bp).

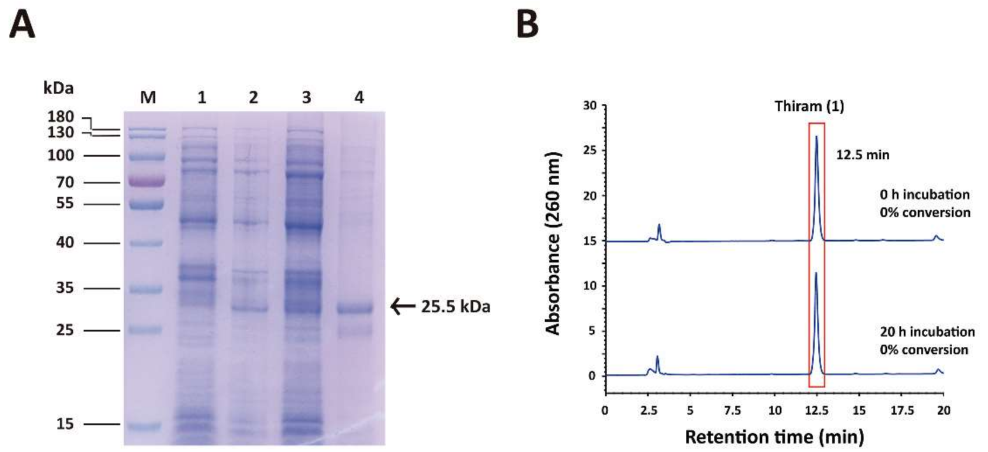

Fig. S17. SDS-PAGE analysis of the lambda class GST from papaya (LCpGST) and activity towards thiram (1). *A*, SDS-PAGE analysis of recombinant LCpGST containing an additional *C*-terminal hexa-histidine tag, after staining with using Coomassie brilliant blue G-250. M: Protein marker; 1: Cell pellets before induction; 2: Cell pellets after induction; 3: Supernatant of cell lysate; 4: Ni-NTA purified enzyme (0.05 µg). *B*, HPLC-based analysis of LCpGST-catalyzed degradation of **1**. The reaction system contained 0.2 mM **1** and 0.01 U LCpGST in 50 mM Tris buffer (total volume = 50 µL), and was shaken at 200 rpm and 28 ºC. The reaction was stopped by adding 50 µL methanol. The degradation of **1** was monitored using an HPLC system equipped with a C18 column at 260 nm. The mobile phase (1 mL/min) consisted of a gradient from 30% to 77% acetonitrile from 0 to 20 min. LCpGST did not degrade **1**. The concentration of **1** decreased with the same rate as in the control experiment, which was carried out in the absence of enzyme.

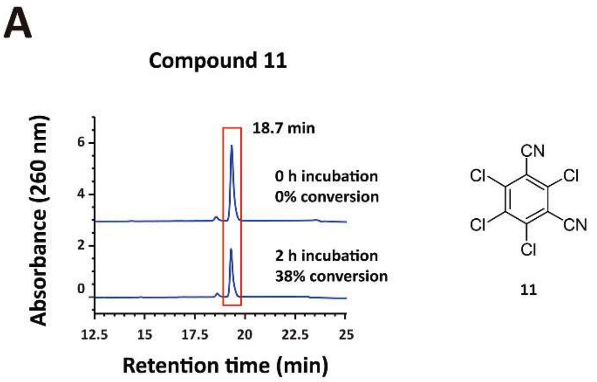

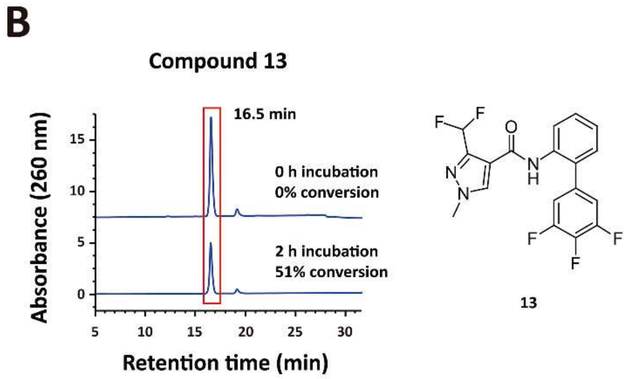

Fig. S18. Catalytic promiscuity of PCpGST against different fungicides commonly used in papaya. *A*, HPLC-based analysis of PCpGST-catalyzed degradation of chlorothalonil (**11**). The reaction system contained 0.2 mM **11** and 0.01 U PCpGST in 50 mM Tris buffer (total volume = 50 μL), and was shaken at 200 rpm and 28 ºC. The reaction was stopped by adding 50 μL methanol. *B*, HPLC-based analysis of PCpGST-catalyzed degradation of fluxapyroxad (**13**). The reaction system contained 0.2 mM **13** and 0.01 U PCpGST in 50 mM Tris buffer (total volume = 50 μL), and was shaken at 200 rpm and 28 ºC. The reaction was stopped by adding 50 μL methanol. The degradation of **11** and **13** was monitored using an HPLC system equipped with a C18 column at 260 nm. The mobile phase (1 mL/min) consisted of a gradient from 30% to 77% acetonitrile from 0 to 20 min.

3. Supplementary tables

Table S1. Predicted GSTs in papaya genome.[a]

GenBank number	Class	Isoform
XP_021891505.1	Lambda	L3
XP_021891574.1	Lambda	L3
**XP_021891575.1** [b]	Lambda	L3
XP_021891502.1	Lambda	L3
XP_021891504.1	Lambda	L3
XP_021891548.1	Lambda	L1
XP_021891501.1	Lambda	L3
XP_021891503.1	Lambda	L3
**XP_021906879.1** [b]	Phi	F6
XP_021895285.1	Theta	T1
XP_021900534.1	Tau	-
XP_021911151.1	Tau	-
XP_021911229.1	Tau	-
XP_021911349.1	Tau	-
XP_021895437.1	Tau	23
XP_021910402.1	Tau	U17
XP_021904662.1	Tau	U9
XP_021897374.1	Tau	-
XP_021897289.1	Tau	-
XP_021896969.1	Tau	-
XP_021896924.1	Tau	-
XP_021896923.1	Tau	-
XP_021895419.1	Tau	-
XP_021895081.1	Tau	-
**XP_021895014.1** [b]	Tau	-
XP_021895003.1	Tau	-

[a] Papaya genome was reported by Ming et al. (4).

[b] The lambda class GST with GenBank number XP_021891575.1, the phi class GST with GenBank number XP_021906879.1, and the tau class GST with GenBank number XP_021895014.1 were used in the experiments.

Table S2. Kinetic parameters of TCpGST towards thiram (**1**) and tetrabenzylthiuram disulfide (**8**) at 28 ºC.

Substrate	K_m (mM)	V_{max} (mM/min)	k_{cat} (min^{-1})	k_{cat}/K_m (mM^{-1} min^{-1})	R^2
**1**	0.1484 ± 0.007	0.1004 ± 0.003	23.602 ± 0.003	159.043	0.9803
**8**	49.769 ± 10.829	42.115 ± 6.820	9900.452 ± 1309.052	198.928	0.9901

Table S3. Nucleotide sequences encoding mutant enzymes.

**Mutations**	
**F10A**	ATGGCGGACGAGGTTGTTCTCTTGGATGCATGGCCAAGCCCTTT<br>TGGAATGAGAATCAGAATCGCTTTAGCCGAGAAGGGTATTCACT<br>ACGAGTACAAGGAAGAGAATCTGAGAAACAAGAGTCCCTTACT<br>CCTGCAGATGAACCCGGTACACAAGAAAATCCCGGTTCTCATC<br>CACAATGGTAAACCCATCTGTGAGTCTTTGATCCAGATTCAGTA<br>CATAGATGAGGTATGGAGCGACAAGGCTCCTCTGCTTCCCTCTG<br>ATCCTTATCAGAGAGCTCAAGCCAGGTTCTGGGCTGACTATGTT<br>GACAAGAAGATGTATGAAGCTGGGAGGAGAGTTTGGACGACTA<br>AAGGGGAAGAACAGGAGGGGGCCAAGAAAGAGTTCATAGAA<br>ATCTTGAAGACTTTGGAGGGAGAACTTGGGGAGAAGCCTTATT<br>TTGGTGGGGAAAGTTTTGGGTATGTGGATTTGACTTTTATCCCAT<br>TCTACACTTGGTTCAGTGTGTATGAAAGTTTTGGGAAGATGAGC<br>ATAGAGGCAGAATGCCCCAAGTTGTTTAGTTGGGTGAAAAGGT<br>GTTTGGAGAAGGAGAGTGTTTCAAAATCTCTGCCTGATCAAGA<br>TAAGGTATACGGCTTCGTTTTGGAACTCAGGAAGGCTCTTGGG<br>ATTTGA
**S13A**	ATGGCGGACGAGGTTGTTCTCTTGGATTTCTGGCCAGCACCTTT<br>TGGAATGAGAATCAGAATCGCTTTAGCCGAGAAGGGTATTCACT<br>ACGAGTACAAGGAAGAGAATCTGAGAAACAAGAGTCCCTTACT<br>CCTGCAGATGAACCCGGTACACAAGAAAATCCCGGTTCTCATC<br>CACAATGGTAAACCCATCTGTGAGTCTTTGATCCAGATTCAGTA<br>CATAGATGAGGTATGGAGCGACAAGGCTCCTCTGCTTCCCTCTG<br>ATCCTTATCAGAGAGCTCAAGCCAGGTTCTGGGCTGACTATGTT<br>GACAAGAAGATGTATGAAGCTGGGAGGAGAGTTTGGACGACTA<br>AAGGGGAAGAACAGGAGGGGGCCAAGAAAGAGTTCATAGAA<br>ATCTTGAAGACTTTGGAGGGAGAACTTGGGGAGAAGCCTTATT<br>TTGGTGGGGAAAGTTTTGGGTATGTGGATTTGACTTTTATCCCAT<br>TCTACACTTGGTTCAGTGTGTATGAAAGTTTTGGGAAGATGAGC<br>ATAGAGGCAGAATGCCCCAAGTTGTTTAGTTGGGTGAAAAGGT<br>GTTTGGAGAAGGAGAGTGTTTCAAAATCTCTGCCTGATCAAGA<br>TAAGGTATACGGCTTCGTTTTGGAACTCAGGAAGGCTCTTGGG<br>ATTTGA
**K53A**	ATGGCGGACGAGGTTGTTCTCTTGGATTTCTGGCCAAGCCCTTT<br>TGGAATGAGAATCAGAATCGCTTTAGCCGAGAAGGGTATTCACT<br>ACGAGTACAAGGAAGAGAATCTGAGAAACAAGAGTCCCTTACT<br>CCTGCAGATGAACCCGGTACACAAGGCAATCCCGGTTCTCATC<br>CACAATGGTAAACCCATCTGTGAGTCTTTGATCCAGATTCAGTA<br>CATAGATGAGGTATGGAGCGACAAGGCTCCTCTGCTTCCCTCTG<br>ATCCTTATCAGAGAGCTCAAGCCAGGTTCTGGGCTGACTATGTT<br>GACAAGAAGATGTATGAAGCTGGGAGGAGAGTTTGGACGACTA<br>AAGGGGAAGAACAGGAGGGGGCCAAGAAAGAGTTCATAGAA

	ATCTTGAAGACTTTGGAGGGAGAACTTGGGGAGAAGCCTTATT
	TTGGTGGGGAAAGTTTTGGGTATGTGGATTTGACTTTTATCCCAT
	TCTACACTTGGTTCAGTGTGTATGAAAGTTTTGGGAAGATGAGC
	ATAGAGGCAGAATGCCCCAAGTTGTTTAGTTGGGTGAAAAGGT
	GTTTGGAGAAGGAGAGTGTTTCAAAATCTCTGCCTGATCAAGA
	TAAGGTATACGGCTTCGTTTTGGAACTCAGGAAGGCTCTTGGG
	ATTTGA
**I54A**	ATGGCGGACGAGGTTGTTCTCTTGGATTTCTGGCCAAGCCCTTT
	TGGAATGAGAATCAGAATCGCTTTAGCCGAGAAGGGTATTCACT
	ACGAGTACAAGGAAGAGAATCTGAGAAACAAGAGTCCCTTACT
	CCTGCAGATGAACCCGGTACACAAGAAAGCACCGGTTCTCATC
	CACAATGGTAAACCCATCTGTGAGTCTTTGATCCAGATTCAGTA
	CATAGATGAGGTATGGAGCGACAAGGCTCCTCTGCTTCCCTCTG
	ATCCTTATCAGAGAGCTCAAGCCAGGTTCTGGGCTGACTATGTT
	GACAAGAAGATGTATGAAGCTGGGAGGAGAGTTTGGACGACTA
	AAGGGGAAGAACAGGAGGGGGCCAAGAAAGAGTTCATAGAA
	ATCTTGAAGACTTTGGAGGGAGAACTTGGGGAGAAGCCTTATT
	TTGGTGGGGAAAGTTTTGGGTATGTGGATTTGACTTTTATCCCAT
	TCTACACTTGGTTCAGTGTGTATGAAAGTTTTGGGAAGATGAGC
	ATAGAGGCAGAATGCCCCAAGTTGTTTAGTTGGGTGAAAAGGT
	GTTTGGAGAAGGAGAGTGTTTCAAAATCTCTGCCTGATCAAGA
	TAAGGTATACGGCTTCGTTTTGGAACTCAGGAAGGCTCTTGGG
	ATTTGA
**E66A**	ATGGCGGACGAGGTTGTTCTCTTGGATTTCTGGCCAAGCCCTTT
	TGGAATGAGAATCAGAATCGCTTTAGCCGAGAAGGGTATTCACT
	ACGAGTACAAGGAAGAGAATCTGAGAAACAAGAGTCCCTTACT
	CCTGCAGATGAACCCGGTACACAAGAAAATCCCGGTTCTCATC
	CACAATGGTAAACCCATCTGTGCATCTTTGATCCAGATTCAGTA
	CATAGATGAGGTATGGAGCGACAAGGCTCCTCTGCTTCCCTCTG
	ATCCTTATCAGAGAGCTCAAGCCAGGTTCTGGGCTGACTATGTT
	GACAAGAAGATGTATGAAGCTGGGAGGAGAGTTTGGACGACTA
	AAGGGGAAGAACAGGAGGGGGCCAAGAAAGAGTTCATAGAA
	ATCTTGAAGACTTTGGAGGGAGAACTTGGGGAGAAGCCTTATT
	TTGGTGGGGAAAGTTTTGGGTATGTGGATTTGACTTTTATCCCAT
	TCTACACTTGGTTCAGTGTGTATGAAAGTTTTGGGAAGATGAGC
	ATAGAGGCAGAATGCCCCAAGTTGTTTAGTTGGGTGAAAAGGT
	GTTTGGAGAAGGAGAGTGTTTCAAAATCTCTGCCTGATCAAGA
	TAAGGTATACGGCTTCGTTTTGGAACTCAGGAAGGCTCTTGGG
	ATTTGA
**S67A**	ATGGCGGACGAGGTTGTTCTCTTGGATTTCTGGCCAAGCCCTTT
	TGGAATGAGAATCAGAATCGCTTTAGCCGAGAAGGGTATTCACT
	ACGAGTACAAGGAAGAGAATCTGAGAAACAAGAGTCCCTTACT
	CCTGCAGATGAACCCGGTACACAAGAAAATCCCGGTTCTCATC
	CACAATGGTAAACCCATCTGTGAGGCATTGATCCAGATTCAGTA

	CATAGATGAGGTATGGAGCGACAAGGCTCCTCTGCTTCCCTCTG<br>ATCCTTATCAGAGAGCTCAAGCCAGGTTCTGGGCTGACTATGTT<br>GACAAGAAGATGTATGAAGCTGGGAGGAGAGTTTGGACGACTA<br>AAGGGGAAGAACAGGAGGGGGCCAAGAAAGAGTTCATAGAA<br>ATCTTGAAGACTTTGGAGGGAGAACTTGGGGAGAAGCCTTATT<br>TTGGTGGGGAAAGTTTTGGGTATGTGGATTTGACTTTTATCCCAT<br>TCTACACTTGGTTCAGTGTGTATGAAAGTTTTGGGAAGATGAGC<br>ATAGAGGCAGAATGCCCCAAGTTGTTTAGTTGGGTGAAAAGGT<br>GTTTGGAGAAGGAGAGTGTTTCAAAATCTCTGCCTGATCAAGA<br>TAAGGTATACGGCTTCGTTTTGGAACTCAGGAAGGCTCTTGGG<br>ATTTGA

Table S4. Kinetic parameters of the mutant enzymes towards thiram (**1**) at 28 ºC.

**Mutation**	K_m **(mM)**	V_{max} **(mM/min)**	k_{cat} **(min**$^{-1}$**)**	k_{cat}/K_m **(mM**$^{-1}$ **min**$^{-1}$**)**	R^2
TCpGST	0.1484 ± 0.007	0.1004 ± 0.003	23.602 ± 0.003	159.043	0.9803
F10A	0.1896 ± 0.0113	0.1128 ± 0.0012	23.866 ± 1.351	125.876	0.9905
S13A	0.1793 ± 0.0076	0.0991 ± 0.0028	23.352 ± 2.896	130.240	0.9827
K53A	0.1977 ± 0.0096	0.0988 ± 0.0072	24.038 ± 1.627	121.588	0.9832
I54A	0.1669 ± 0.0161	0.1062 ± 0.0101	21.695 ± 2.504	129.988	0.9698
E66A	0.1462 ± 0.0235	0.1015 ± 0.0049	22.169 ± 1.713	151.634	0.9726

Table S5. Kinetic parameters of the mutant enzymes towards glutathione at 28 ºC.

Mutation	K_m (mM)	V_{max} (mM/min)	k_{cat} (min^{-1})	k_{cat}/K_m (mM^{-1} min^{-1})	R^2
TCpGST	0.0300 ± 0.0083	0.5522 ± 0.0045	11.7904 ± 1.016	393.015	0.9691
F10A	0.0896 ± 0.0163	0.1278 ± 0.0211	24.733 ± 3.251	276.035	0.9853
S13A	0.0793 ± 0.0189	0.0089 ± 0.0007	2.6962 ± 0.456	34.000	0.9903
K53A	0.0477 ± 0.0115	0.0621 ± 0.0076	14.0265 ± 1.717	294.058	0.9756
I54A	0.144 ± 0.0488	0.1271 ± 0.0236	29.6423 ± 5.504	205.849	0.9844
E66A	0.0698 ± 0.008	0.0795 ± 0.004	18.099 ± 0.911	191.316	0.9875
S67A	0.0896 ± 0.0163	0.1278 ± 0.0211	24.733 ± 3.251	276.035	0.9880

Table S6. Kinetic parameters of PCpGST towards thiram (**1**) at 28 ºC.

**Mutation**	K_m **(mM)**	V_{max} **(mM/min)**	k_{cat} **(min^{-1})**	k_{cat}/K_m **(mM^{-1} min^{-1})**	R^2
PCpGST	0.0780 ± 0.0170	0.1095 ± 0.0119	4741.8 ± 72.8	60,788.5	0.9823

Table S7. PCR reaction system for *TCpGST*, *PCpGST*, and *LCpGST* amplification.

**Reagents**	**Volume (µL)**
PrimerSTAR Max Premix	25
DNA template	1
Forward primer	1
Reverse primer	1
H_2O	22

Table S8. Nucleotide and amino acid sequences of TCpGST, PCpGST, and LCpGST.

**TCpGST**
**Amino acid sequence**
MADEVVLLDFWPSPFGMRIRIALAEKGIHYEYKEENLRNKSPLLLQMNPVH KKIPVLIHNGKPICESLIQIQYIDEVWSDKAPLLPSDPYQRAQARFWADYVD KKMYEAGRRVWTTKGEEQEGAKKEFIEILKTLEGELGEKPYFGGESFGYVD LTFIPFYTWFSVYESFGKMSIEAECPKLFSWVKRCLEKESVSKSLPDQDKVY GFVLELRKALGI
**Nucleotide (cds) sequence**
ATGGCGGACGAGGTTGTTCTCTTGGATTTCTGGCCAAGCCCTTTTGGAAT GAGAATCAGAATCGCTTTAGCCGAGAAGGGTATTCACTACGAGTACAAG GAAGAGAATCTGAGAAACAAGAGTCCCTTACTCCTGCAGATGAACCCGG TACACAAGAAAATCCCGGTTCTCATCCACAATGGTAAACCCATCTGTGAG TCTTTGATCCAGATTCAGTACATAGATGAGGTATGGAGCGACAAGGCTCC TCTGCTTCCCTCTGATCCTTATCAGAGAGCTCAAGCCAGGTTCTGGGCTG ACTATGTTGACAAGAAGATGTATGAAGCTGGGAGGAGAGTTTGGACGAC TAAAGGGGAAGAACAGGAGGGGGCCAAGAAAGAGTTCATAGAAATCTT GAAGACTTTGGAGGGAGAACTTGGGGAGAAGCCTTATTTGGTGGGGAA AGTTTTGGGTATGTGGATTTGACTTTTATCCCATTCTACACTTGGTTCAGT GTGTATGAAAGTTTTGGGAAGATGAGCATAGAGGCAGAATGCCCCAAGT TGTTTAGTTGGGTGAAAAGGTGTTTGGAGAAGGAGAGTGTTTCAAAATC TCTGCCTGATCAAGATAAGGTATACGGCTTCGTTTTGGAACTCAGGAAGG CTCTTGGGATTTGA
**PCpGST**
**Amino acid sequence**
MADEVVLLDFWPSPFGMRIRIALAEKGIHYEYKEENLRNKSPLLLQMNPVH KKIPVLIHNGKPICESLIQIQYIDEVWSDKAPLLPSDPYQRAQARFWADYVD KKMYEAGRRVWTTKGEEQEGAKKEFIEILKTLEGELGEKPYFGGESFGYVD LTFIPFYTWFSVYESFGKMSIEAECPKLFSWVKRCLEKESVSKSLPDQDKVY GFVLELRKALGI
**Nucleotide (cds) sequence**
ATGGCGGACGAGGTTGTTCTCTTGGATTTCTGGCCAAGCCCTTTTGGAAT GAGAATCAGAATCGCTTTAGCCGAGAAGGGTATTCACTACGAGTACAAG GAAGAGAATCTGAGAAACAAGAGTCCCTTACTCCTGCAGATGAACCCGG TACACAAGAAAATCCCGGTTCTCATCCACAATGGTAAACCCATCTGTGAG TCTTTGATCCAGATTCAGTACATAGATGAGGTATGGAGCGACAAGGCTCC TCTGCTTCCCTCTGATCCTTATCAGAGAGCTCAAGCCAGGTTCTGGGCTG ACTATGTTGACAAGAAGATGTATGAAGCTGGGAGGAGAGTTTGGACGAC TAAAGGGGAAGAACAGGAGGGGGCCAAGAAAGAGTTCATAGAAATCTT GAAGACTTTGGAGGGAGAACTTGGGGAGAAGCCTTATTTGGTGGGGAA AGTTTTGGGTATGTGGATTTGACTTTTATCCCATTCTACACTTGGTTCAGT GTGTATGAAAGTTTTGGGAAGATGAGCATAGAGGCAGAATGCCCCAAGT TGTTTAGTTGGGTGAAAAGGTGTTTGGAGAAGGAGAGTGTTTCAAAATC

TCTGCCTGATCAAGATAAGGTATACGGCTTCGTTTTGGAACTCAGGAAGG CTCTTGGGATTTGA
**LCpGST**
**Amino acid sequence**
MATGAEEDLPLPLDATAEQPNLFDGTTRLYTCYTCPYAQRVWITRNYKGLE DEIKLVPLNLRNRPAWYKEKVYSLNKVPSLEHNGKVMGESLDLIKYVDSNF RGPSLLPDDPTKREFAEEMLRYTDTFNMAVYISLNGDAVKESGPSFDYLENA LHKFDDGPFFLGGFSLVDIAYIPFVERFQIFFADVWNYDITESRPKLATWIEV
**Nucleotide (cds) sequence**
ATGGCTACTGGTGCGGAAGAGGATTTGCCATTGCCACTAGATGCTACAGC GGAGCAGCCAAATCTCTTTGATGGAACCACAAGGTTATATACTTGTTACA CGTGTCCATATGCGCAACGTGTGTGGATCACAAGGAATTACAAGGGATTA GAAGACGAGATTAAATTGGTTCCTCTAAACCTCCGGAACAGGCCTGCAT GGTATAAGGAGAAAGTATACTCCCTAAATAAGGTTCCATCATTGGAACAC AATGGCAAAGTCATGGGGGAGAGTCTTGACTTGATCAAGTACGTAGACA GTAACTTTCGGGGGCCTTCCCTTTTACCTGATGATCCTACCAAAAGAGAA TTTGCTGAAGAGATGCTTAGATATACTGATACATTTAACATGGCCGTGTAT ATTTCACTTAATGGAGATGCTGTGAAAGAATCTGGACCTTCTTTTGATTAC TTAGAAAATGCCCTGCATAAATTTGACGATGGCCCATTCTTCCTTGGTGG ATTTAGCTTGGTGGATATAGCTTATATTCCATTTGTCGAGAGATTCCAAATC TTTTTTGCAGATGTATGGAATTATGACATAACAGAAAGCAGGCCTAAACT AGCAACATGGATTGAGGTT

Table S9. Energy parameters of the docking model of thiram in TCpGST.[a]

Parameter	Value
Energy	-20.4182
SimpleFitness	-20.4182
FullFitness	-1437.71
InterFull	-30.2222
IntraFull	-10.1001
solvFull	-1575.14
surfFull	177.757
extraFull	0
deltaGcompsolvpol	-1575.14
deltaGprotsolvpol	177.757
deltaGprotsolvnonpol	-1582.1
deltaGligsolvpol	179.511
deltaGligsolvnonpol	-3.84073
deltaGvdw	5.30034
deltaGelec	-30.2222
deltaG	0
Cluster	-6.85536
ClusterRank	51

[a] The interactions and distances between thiram and the residues of TCpGST active site were calculated using SwissDock.

Table S10. Primers for PCR site-directed mutation.

Mutation site	Primer	Primer sequence
F10	Forward	CTTGGATGCATGGCCAAGCCCTTTTGGA
	Reverse	TTGGCCATGCATCCAAGAGAACAACCTCGTC
S13	Forward	TCTGGCCAGCACCTTTTGGAATGAGAATCAGAATCG
	Reverse	AAAAGGTGCTGGCCAGAAATCCAAGAGAACAA
K53	Forward	TACACAAGGCAATCCCGGTTCTCATCCACAAT
	Reverse	CGGGATTGCCTTGTGTACCGGGTTCATCTGCA
I54	Forward	CAAGAAAGCACCGGTTCTCATCCACAATGGTA
	Reverse	GAACCGGTGCTTTCTTGTGTACCGGGTTCATCT
E66	Forward	CCATCTGTGCATCTTTGATCCAGATTCAGTACATAGATG
	Reverse	CAAAGATGCACAGATGGGTTTACCATTGTGGA
S67	Forward	CTGTGAGGCATTGATCCAGATTCAGTACATAGATGAGG
	Reverse	GGATCAATGCCTCACAGATGGGTTTACCATTGTG

Table S11. PCR reaction system for site-directed mutation.

Reagents	Volume (μL)
PrimerSTAR Max Premix	25
DNA template	0.2
Forward primer	1
Reverse primer	1
H_2O	22.8

Table S12. LC-MS operating conditions.

**Negative mode**
Curtain Gas (CUR): 30
IonSpray Voltage (IS): –4500
Temperature (TEM): 550
Ion Source Gas 1 (GS1): 55
Declustering Potential (DP): –98
Entrance Potential (EP): –10
Collision Energy (CE): –20
Collision Cell Exit Potential (CXP): –11.5
**Positive mode**
Curtain Gas (CUR): 30
IonSpray Voltage (IS): 4500
Temperature (TEM): 550
Ion Source Gas 1 (GS1): 55
Declustering Potential (DP): 100
Entrance Potential (EP): 10
Collision Energy (CE): 27
Collision Cell Exit Potential (CXP): 11.5

Table S13. GC-MS operating conditions for the detection of carbon disulfide (**4**).

**Gas chromatography analysis**
**Oven temperature:** 40 ºC (3 min) → 50 ºC/min until 240 ºC (5 min)
**Carrier gas:** helium, 40 cm/s
**Split flow ratio:** 50/1
**Injection volume:** 1 mL
**Column:** DB-17 ms (film thickness: 0.25 µm, 30 m × 250 µm)
**Ion source temperature:** 230 ºC (EI)
**Ionization volt:** 70 eV
**Mass range:** 60-250 *m/z*
**Mode:** full scan
**Quantitative ion:** C=S: *m/z* 44; CS_2: *m/z* 76

Table S14. Primers used in the qRT-PCR analysis.

Gene	Forward primer	Reverse primer
Actin	AGGCAGGCAAGAGAAGAT	TTCATACCGAGTAGCGATTC
TCpGST	ATGGCGGACGAGGTTGTTCTC	TCAAATCCCAAGAGCCTTCCTGAG

References

1. Basulto, F. S., Duch, E. S., Espadas y Gil, F., Plaza, R. D., Saavedra, A. L., and Santamaria, J. M. (2009). Postharvest ripening and maturity indices for maradol papaya. *Interciencia* **34**, 583–588
2. Tamura, K., Stecher, G., and Kumar, S. (2021). MEGA11 molecular evolutionary genetics analysis version 11. *Mol. Biol. Evol.* **38**, 3022–3027
3. Jo, H. J., Kong, J. N., Lim, J. K., and Kong, K. H. (2014). Site-directed mutagenesis of evolutionarily conserved serine residues in the *N*-terminal domain of rice phi-class glutathione *S*-transferase F5. *J. Mol. Catal. B Enzym.* **106**, 71–75
4. Ming, R., Hou, S. B., Feng, Y., Yu, Q. Y., Dionne-Laporte, A., Saw, J. H., *et al.* (2008). The draft genome of the transgenic tropical fruit tree papaya (*Carica papaya* Linnaeus). *Nature* **452**, 991–997
